# PACo: A Novel Procrustes Application to Cophylogenetic Analysis

**DOI:** 10.1371/journal.pone.0061048

**Published:** 2013-04-08

**Authors:** Juan Antonio Balbuena, Raúl Míguez-Lozano, Isabel Blasco-Costa

**Affiliations:** 1 Cavanilles Institute of Biodiversity and Evolutionary Biology, University of Valencia, Valencia, Spain; 2 Institute of Parasitology, Academy of Sciences of the Czech Republic, České Budějovice, Czech Republic; Field Museum of Natural History, United States of America

## Abstract

We present Procrustean Approach to Cophylogeny (PACo), a novel statistical tool to test for congruence between phylogenetic trees, or between phylogenetic distance matrices of associated taxa. Unlike previous tests, PACo evaluates the dependence of one phylogeny upon the other. This makes it especially appropriate to test the classical coevolutionary model that assumes that parasites that spend part of their life in or on their hosts track the phylogeny of their hosts. The new method does not require fully resolved phylogenies and allows for multiple host-parasite associations. PACo produces a Procrustes superimposition plot enabling a graphical assessment of the fit of the parasite phylogeny onto the host phylogeny and a goodness-of-fit statistic, whose significance is established by randomization of the host-parasite association data. The contribution of each individual host-parasite association to the global fit is measured by means of jackknife estimation of their respective squared residuals and confidence intervals associated to each host-parasite link. We carried out different simulations to evaluate the performance of PACo in terms of Type I and Type II errors with respect to two similar published tests. In most instances, PACo performed at least as well as the other tests and showed higher overall statistical power. In addition, the jackknife estimation of squared residuals enabled more elaborate validations about the nature of individual links than the ParaFitLink1 test of the program ParaFit. In order to demonstrate how it can be used in real biological situations, we applied PACo to two published studies using a script written in the public-domain statistical software R.

## Introduction

The phenomenal growth in sequence information in the last decades has propelled the development of phylogenetic approaches to ecology and evolution. Aimed at understanding coevolutionary and cospeciation processes, cophylogeny focuses on species associations (organisms tracking organisms, such as parasites and hosts or pollinators and flowering plants) [1], [2], molecular systematics (organisms or genes tracking genes) [3], [4] and historical biogeography (organisms tracking areas) [5], [6]. Cophylogenetic studies stem from the observation that the diversification patterns over evolutionary time of tightly associated organisms, such as parasites and their hosts, are seldom independent [2]. Thus some degree of topological similarity, often termed congruence [7], between the phylogenies of the associated taxa is expected to occur. Congruence quantifies the extent to which each node in a given tree maps to a corresponding position in the other tree and perfect congruence can be interpreted as evidence for cospeciation, which may or may not result from coevolutionary mechanisms [8], [9]. Such perfect congruence is rarely, if ever, observed in nature, because in addition to cospeciation, three other types of evolutionary events can act concurrently, namely host-switching (the parasite is able to colonize a new unrelated host), duplication (independent speciation of the parasite), and lineage sorting (failure to speciate or disappearance of a parasite linage on a host lineage) [10], [11]. (For simplicity, the evolutionary events are presented and discussed herein in the context of host-parasite systems, but they can be readily adapted and generalized to any other cophylogenetic scenario). Thus, the historical reconstruction of the associations between two given sets of organisms is not straightforward because it needs to evaluate and disentangle the relative roles played by all four evolutionary processes.

The numerous methods of cophylogenetic analysis currently available can be broadly classified in two categories: event-based methods and global-fit methods [12]. The former are aimed at finding the most probable coevolutionary history of the associated taxa. Numerous approaches, based on character optimization, e.g. Brooks’ Parsimony Analysis [13], tree reconciliation of the associated taxa, e.g. COMPONENT [14] and PACT [6], or assignment of relative costs to the evolutionary events, e.g., TreeMap [15], Jungles [16], Tarzan [17] and Jane [18], have been proposed. Event-based methods have strong appeal because they promise to deliver the coevolutionary history of the associated taxa. However, the challenges faced in their application are important. First, well resolved phylogenies are required to obtain reliable results and even with a small number of taxa the number of equally parsimonious solutions can be exceedingly high [12], [19]. Second, event-cost methods are strongly dependent on a good estimation of the set of costs considered [20]. Third, given that not all the topological congruence between trees is necessarily a result of cospeciation [21], the precise reconstruction of coevolutionary history often requires additional data, such as the ages of the nodes, assumptions on the probability of the different events, consideration to the geological history of the areas involved and experimental evidence, such as reciprocal transplant experiments [8], [22].

For their part, global-fit methods are used to quantify the degree of congruence between two given topologies, and identify the associations contributing to the cophylogenetic structure. Although they do not explicitly evaluate evolutionary scenarios, the amount of phylogenetic congruence can be related to the importance of coevolution in the system studied [12]. In addition, there is a clear need for this kind of methods because they afford large-scale cophylogenetic analyses for which the application of event-based counterparts becomes computationally prohibitive [23], [24]. To some extent, the approach taken by global-fit methods is similar to statistical tests for congruence between two given trees. A large variety of approaches have been proposed for this problem, e.g. [25], [26], [27], [28], including a Procrustes-based technique [29] similar to the one described herein. Even methods based on maximum likelihood and Bayesian inference have been specifically designed to study the cophylogeny of host and parasites [30]. However, the applicability of these methods to cophylogenetic studies is limited because they are primarily intended for one-to-one associations, something that rarely occurs in nature [24], [31], [32], [33].

Among the several of global-fit methods currently available, e.g. [7], [34], [35], ParaFit [7] has been the most used one, e.g. [3], [24], [36], [37]. ParaFit is an application to a phylogenetic context of the 4th-corner problem [38], testing whether or not the topological position of parasites in a tree is independent from the phylogenetic position of the associated hosts. The test requires three data matrices as input. The first one is a presence/absence matrix describing the host-parasite associations, whereas the two others contain information of the phylogenetic trees of hosts and parasites. Usually they consist of pairwise patristic or genetic distances, which are transformed into principal coordinate (PCo) matrices. The host PCo matrix is transposed and the three matrices (transposed host PCo, host-parasite association and parasite PCo matrices) are combined into a new one, whose trace is used to obtain a global goodness-of-fit statistic of congruence between the two trees. The significance of the statistic is established by randomization of the host-parasite association matrix. ParaFit also provides two statistics (ParaFitLink1 and 2) for testing individual host-parasite links using similar randomization procedures [7].

A second, more recent, test was proposed by Hommola et al. [34]; for convenience it will be hereafter referred to as HCT for Hommola et al. Cospeciation Test. HCT is a generalization of the Mantel test that correlates the host and parasite phylogenetic distance matrices accommodating multiple hosts associated to a single parasite and vice versa. The method is based on composing a host and a parasite vector using the patristic or genetic distances between the taxa and computing a correlation coefficient between the vectors. Unlike ParaFit, this method does not evaluate the contribution of individual host-parasite links to the global cophylogenetic structure. In addition, HCT differs from ParaFit in the randomization procedure to test the significance of the global-fit statistic. In HCT the null hypothesis is that the host and parasite phylogenies are unrelated. So the labels of the host and parasite phylogenies are randomly and separately permuted, while the tree topologies and host-parasite association matrix remain unchanged. In ParaFit, the null hypothesis states that the parasites species are randomly associated to leaves of the host phylogenetic trees and significance is established by randomization of the host-parasite matrix.

In this paper we introduce PACo (**P**rocrustes **A**pproach to **Co**phylogeny) – a new test based on Procrustes analysis. Procrustes analysis is an extremely flexible technique used for displaying two or more multivariate datasets in their optimal superimposition [38]. Our method provides a superimposition plot enabling a graphical comparison of the fit of the host-parasite associations. In addition, residual analysis affords evaluating the contribution of each individual host-parasite associations to the global fit. Like ParaFit and HCT, PACo is a distance-based test that can be carried out with any pair of distance or dissimilarity matrices, i.e., fully resolved host and parasite phylogenies are not required, and allows for multiple host-parasite associations and different number of hosts and parasites. For this purpose, rows of the host and parasite matrices are replicated to account for the multiple host-parasite links. PACo is also similar to ParaFit in that it uses the same three data matrices as input and converts the phylogenies to PCo coordinates, and it is possible to assess the contribution of individual host-parasite associations to the global topological congruence.

An important conceptual difference with the previous tests is that both ParaFit and HCT compare the host and parasite distance matrices and test for random association between the host and parasite taxa, whereas PACo explicitly tests the dependence of the parasite phylogeny upon the host phylogeny, because in the Procrustean superimposition, the parasite matrix is rotated and scaled to fit the host matrix. Accordingly the permutational procedure to test for global significance of the fit is also different by assigning hosts randomly to parasites. PACo is appropriate to establish whether the classical view of host-parasite cospeciation, which assumes that parasite speciation is driven by host speciation [2], [39], holds in a given host-parasite system. Thus the null hypothesis tested is different from that of ParaFit and HCT, although sufficiently similar as to justify a comparison of the three methods.

In the present study, we carried out several simulation experiments to compare the performance of the new test with that of ParaFit and HCT in terms of Type I and Type II errors. An additional recent test for congruence between phylogenetic trees [35] requires ultrametric trees and, given the complexity of the algorithm, detailed comparison with PACo deserves separate attention. Thus, the present study is restricted to the analysis of additive trees. We show herein that, in most cases, PACo performs at least as well as ParaFit and HCT, and in some instances, it produces better Type I errors that ParaFit and higher statistical power than ParaFit and HCT. Finally, the use of the new test is demonstrated with a script written in the public-domain statistical software R applied to two case studies [32], [40] that illustrate how the residuals of the Procustean fit can give further insight into the nature of individual taxa associations.

## Materials and Methods

### PACo Analysis

**Figure 1:**
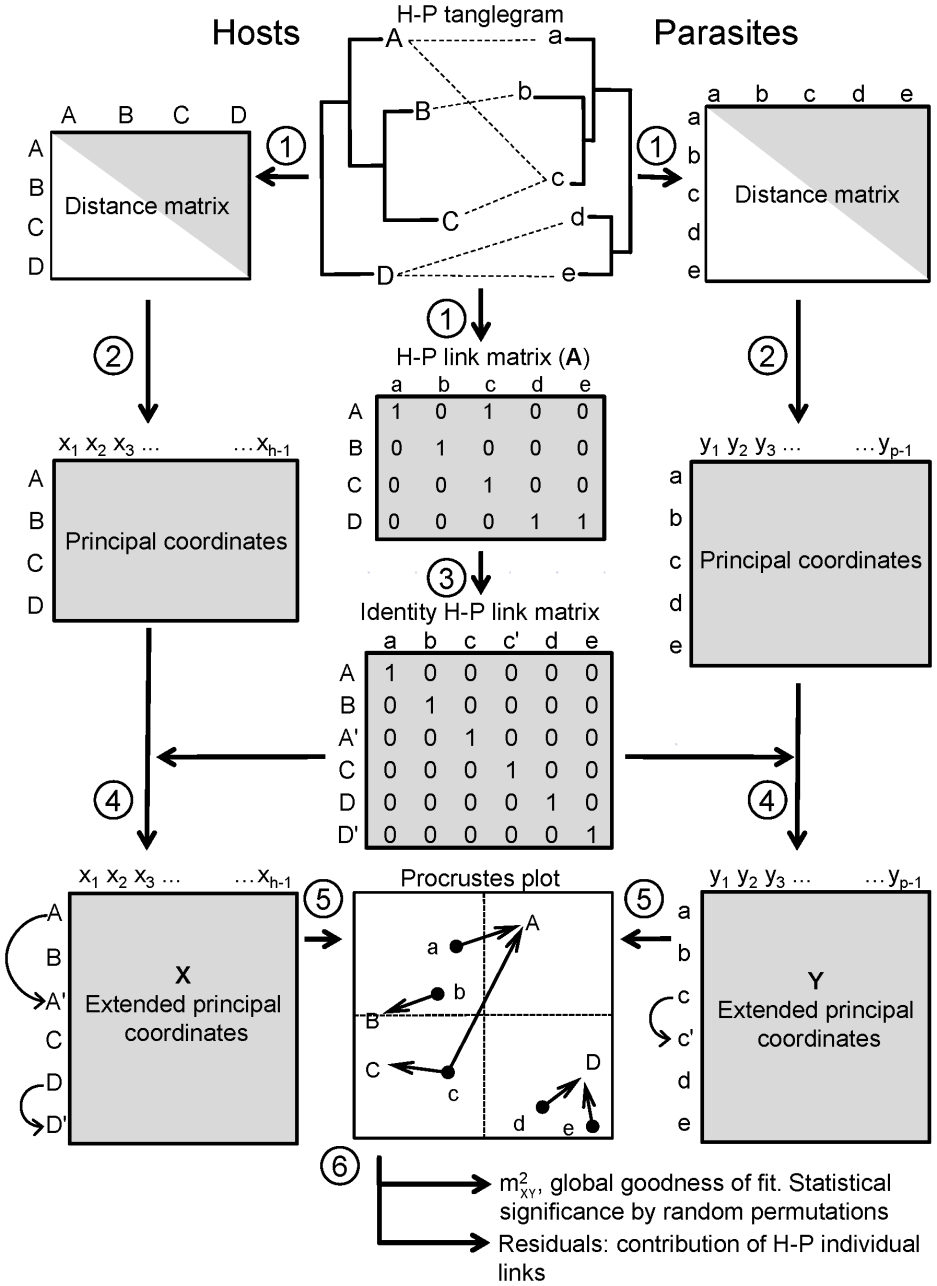
Method overview of PACo. (1) The phylogenetic information encapsulated by the host-parasite (H-P) tanglegram gives way to two distance matrices of host and parasites, and a binary matrix of host-parasite (H-P) links. (2) The distance matrices are transformed by Principal Coordinates. (3) The H-P link matrix (**A**) is converted into an identity matrix to account for multiple host-parasite associations. (4) Rows in the Principal Component matrices are duplicated (arched arrows) following the order dictated by the identity matrix. (5) The extended Principal Coordinate matrices (**X** and **Y**) are centred by mean column vectors and subjected to Procrustes analysis, where the parasite configuration is rotated and scaled to fit the host configuration. The fit can be visualised in a Procrustes superimposition plot. (6) The analysis yields a global goodness-of-fit statistic (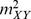), whose significance can be established by a randomization procedure, and individual link residuals that can be further analysed to establish the contribution of each H-P link to the global fit.

The present test builds on three pieces of information: two phylogenetic trees corresponding to hosts and parasites, and a binary matrix (**A**) coding the host-parasite associations (Fig. 1). Let *h* and *p* be the numbers of host and parasite species in the respective phylograms, **A** is an *h* × *p* matrix, where 1 denotes presence of a given parasite species in a given host species, and 0 corresponds to absence of a particular parasite species in a particular host species (Fig. 1). [Note the arbitrary assignation of hosts to rows and parasites to columns. Although the original ParaFit test of Legendre et al. [7] and HCT use **A′**, we opted to adopt the same input format required for the parafit function of the ape package of R [41] to ease comparison and integration with our R script implementing PACo.] The R code needed and instructions to implement PACo in R are given in File S1. In addition, an annotated code version, the input file examples and R code for the simulations described below can be downloaded at http://www.uv.es/cophylpaco/index.html.

Figure 1 provides an overview of how PACo works. First, the host and parasite phylogenies are transformed into their respective distance matrices between species. This can be achieved by computing either patristic or genetic distances, or any dissimilarity measure between the species involved. The host and parasite distance matrices are, in turn, transformed into their respective matrices of principal coordinates (PCo), with *h* and *p* rows, and *h* –1 and *p* –1 columns, the latter representing each of the PCo axes. The PCo matrices can be viewed as representations of the host and parasite phylogenies in a Euclidean hyperspace, although they may contain noisy information with respect to the true phylogeny [7], [42].

PACo contemplates a given parasite occurring in more than one host species and, conversely, a host harbouring more than one parasite species (Fig. 1). Since Procrustes analysis requires the same number of observations in both ordinations, **A** is transformed into an identity matrix by duplicating multiple associations, which in turn are used to replicate in the right order rows of hosts harbouring more than a parasite (PCo hosts) and the corresponding parasites occurring in more than one host (PCo parasites, see Fig. 1). It has been shown in studies using the Mantel test that the replication of taxa produces incorrect Type I rates [34]. Although we had no sufficient a priori information on the behaviour Procrustes analysis with duplicated data points, we show below through simulations that no systematic biases in *P* values were produced and the Type I errors were mostly correct (see below). This is probably so because the replicated taxa in the corresponding PCo matrices are treated as independent observations occupying identical positions in the hyperspace. Next, the expanded matrices of PCo coordinates of hosts (**X**) and parasites (**Y**), with column vectors centred on their respective means, are compared by means of Procrustes analysis using least-squares superimposition. Whereas the **X** configuration is kept fixed, the **Y** counterpart is scaled, centred, mirrored (if necessary) and rotated to minimize the squared differences between the two configurations [43], [44]. If **X** and **Y** do not contain the same number of columns, the narrow matrix is completed with the appropriate number of zero columns. The Procrustean fit of **Y** onto **X** can be visualised in an ordination plot (Fig. 1) and yields a residual sum of squares 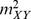, which is computed as follows:
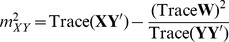
(1)where **W** is obtained by singular value decomposition of (**X′Y**) = **VWU′** [38]. Given that 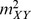 is inversely proportional to the topological congruence between the two ordinations, it represents a measure of the fit of the parasite phylogeny onto the host phylogeny. Note that the statistic is asymmetric, i.e. 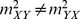. (Not to be confused with the nature of the Procrustean fit, which itself can be symmetric or asymmetric [43]). It is possible to obtain a symmetric statistic by normalizing the column vectors of **X** and **Y** [44], [45]. This approach yields a dimensionless residual sum of squares, which is appropriate in an ecological context [45] where the original variables have different units. Herein, we adopted the asymmetric 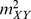
because the PCo axes taken all together preserve the original dissimilarities among the taxa [46] and thus it provides a goodness-of-fit statistic with squared units of the original dissimilarity measure of the host phylogeny. In addition, some of our preliminary analyses using the symmetric sum of squares yielded biased Type I errors perhaps due to the influence of the replicated taxa on the estimated variances computed for normalization of the column vectors of **X** and **Y**.

### Goodness-of-fit Test

The global fit of the regression of the parasite phylogeny onto the host phylogeny can be tested taking 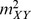 as a test statistic whose significance is established by a randomization procedure. Since **A** encapsulates the associations between hosts and parasites, it is the element that can be randomized under different criteria for hypothesis testing [7], [38], [45]. Given that in PACo we specifically test whether the parasite phylogeny depends on the host phylogeny, hosts are randomly allocated to parasites (i.e., each row in **A** is permuted independently). Thus, the null hypothesis (*H_0_*) is that the host ordination does not predict the parasite ordination and so the parasite clades are randomly associated to the host clades. Conversely, the alternative hypothesis (*H_1_*) implies that at least some part of the parasite ordination is constrained by that of the hosts and, thus the host-parasite associations are to some extent mirrored in phylogenetic congruence.

Testing *H_0_* against *H_1_* with PACo involves the following steps [27]:

1. Set the desired significance level *α*.
2. Compute the observed 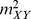 using Equation 1.
3. Obtain a randomized host-parasite association matrix **Z** by permuting the rows of **A** independently. Compute the new statistic 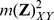 as in step 2, with **Z** instead of **A**.
4. Repeat step 3 a large number of times and keep each 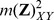 for further reference.
5. Estimate the one-tailed probability *P* of the data under *H_0_* as the proportion of 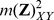 values ≤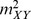. If *P*≤ *α*, *H_0_* can be rejected and the analysis provides evidence for significant dependence of the parasite phylogeny on the host phylogeny.

### Simulations

In any hypothesis test, two kinds of errors can be committed: *H_0_* can be rejected when *H_0_* is true (Type I error) or *H_0_* can be accepted when *H_0_* is false (Type II error) [47]. In order to estimate and compare both the Type I and Type II error rates obtained with ParaFit, HCT and PACo, we carried out several simulation experiments. For each simulation, exactly the same data (i.e., hosts and parasite phylogenetic trees, and **A**) were used, thus rendering the results directly comparable between the three tests. All simulations were carried out with R 2.14.1 [48]. Random additive phylogenetic trees were generated with the function rtree of the ape package [41] with branch lengths drawn randomly from the uniform distribution. The ParaFit global test [7] was carried out with the parafit function of ape, and HCT and PACo were implemented, respectively, with a script by K. Hommola (available at http://www1.maths.leeds.ac.uk/~kerstin/. Accessed 2013 March 11.) and our script based on the procrustes function of the vegan package [43] (File S1). Given that phylogenetic distances are often non-Euclidean [42], the transformation to PCo coordinates may produce negative eigenvalues, whose axes cannot be represented on the real space. To avoid this problem, the Cailliez correction [49] was used as default in the simulations with both PACo and ParaFit. Although this approach may inflate the total sum of squares [44], [50], it did not result in any substantial decrease in the Type I error as shown in the results below.

#### Type I error

For a test to be correct, the probability of committing a Type I error should not exceed the nominal significance level of the test *α*. In order to estimate the Type I errors of the three tests, we simulated data under *H_0_*. In each simulation, a pair of random host and parasite trees, and a corresponding **A** containing a random sample (without replication) of all possible of parasite links were generated. The following parameter combinations were used in the simulations:

- 10 hosts, 10 parasites, and 10, 15, 20 and 25 host-parasite random links.
- 10 hosts, 15 parasites, and 10, 15, 20 and 25 links.
- 15 hosts, 10 parasites, and 10, 15, 20 and 25 links.
- 20 hosts, 20 parasites, and 20, 25, 30 and 35 links.

To our knowledge this is the first time that Type I errors of ParaFit and HCT are evaluated with larger phylogenies (>15 taxa) as in (d), which is of practical interest given the current availability of phylogenies of this size range.

For each parameter combination, 10,000 simulations were generated and the *P* values were calculated based on 999 permutations for each method in each simulation. For each set of simulations, the correctness of the Type I errors was evaluated by two procedures: (1) Type I error rates were computed for the commonly used 0.01 and 0.05 significance levels, together with their 95% confidence intervals based on 1,000 bootstrap samples of the 10,000 simulations. (2) To evaluate the overall accuracy of the error rates for any significance level, plots of the empirical cumulative distribution function of the *P* values resulting from each parameter combination were composed. When *H_0_* is true, correctly formed *P* values must follow a uniform distribution (i.e., *y* = *x*) [24].

#### Type II error

We assessed the Type II error rate as the statistical power of the test, which is measured as the probability of rejecting a false *H_0_*. The power of the three tests was estimated and compared through simulations where *H_0_* was made to be false by construct. Three types of simulations, adapted from Legendre et al. [7], were performed:

1. *Random links added.* In each simulation, a single random tree was generated to represent identical phylogenies for host and parasites. Then **A** was formed by associating each host species to the parasite species at the corresponding position on the tree. These host-parasite systems could be viewed as representing ideal coevolutionary scenarios. Next a given number of random host-parasite links was added to **A** without replacing the existing links. Simulations were carried out with 10 hosts and 10 parasites and with 20 hosts and 20 parasites, with a number of added random links equal to 0%, 20%, 40%, 60%, 80% and 100% of the number of coevolutionary links.
2. *Coevolutionary links replaced.* The host and parasite trees, and **A** were generated as in the previous set of simulations. Then a given number of coevolutionary links in **A** was replaced (without replication of existing links) by an equal number of randomly located links. The following parameter combinations were explored: 10 hosts and 10 parasites, and 20 hosts and 20 parasites, replacing 0%, 20%, 40%, 60%, 80% and 100% of the number of coevolutionary links.
3. *Partly congruent trees.* In this set of simulations, a portion of the host and parasite trees was identical, whereas the remainder was generated at random. Then, coevolutionary links were created between host and parasites placed in the common part of the tree, whereas hosts and parasites in the random part of the tree were related by random links. Simulations were carried out with 10 hosts, 10 parasites and 10 host-parasite links, and with 20 hosts, 20 parasites and 20 host-parasite links, with varied proportions of coevolutionary links: 100%, 80%, 60%, 40%, 20% and 0% of the total number of links.

We applied the three tests to 10,000 simulations for each of these parameter combinations. Statistical power was estimated, based on 999 permutations for each method in each simulation, as the rejection rate of the false *H_0_* at the 0.01 and 0.05 significance levels.

### Contribution of Individual Links

PACo is amenable to statistically testing the significance of the individual links. For instance, an analogue to ParaFitLink1 of Legendre et al. [7] can be devised by replacing with 0 the value 1 of the *i* ^th^ link representing a host-parasite link in **A**. A new sum of squared residuals can then be estimated for the *i* ^th^ link and the significance of the difference between the new statistic and 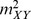 can be established by random permutations. However, we did not pursue this approach because multiple testing of the host-parasite links requires adjusting the *α* levels to account for the increased Type I error rates. Although there are procedures to correct for this effect [51], it comes at the cost of reducing statistical power [52], [53], [54]. Since finding the appropriate adjustment of α can be very complex, we propose a strategy based on assessing the biological relevance [55] of each host-parasite link contributing to the global fit. Given that 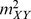 represents the sum of squared residuals of each link 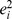, the latter provides a direct measure of host-parasite link importance. The 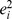
’s, together with their 95% confidence intervals, can be estimated using a jackknife method [47] as follows:

1. Compute 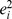 for each of the *n* links.
2. For *i* = 1 to *i* = *n*,
  1. replace the value 1 in **A** corresponding to the *i* ^th^ link with 0, to yield a new host-parasite association matrix **A**(-*i*).
  2. For *j* = 1 to *j* = *n*; if *j* ≠ *i* then
    1. estimate the *n* –1 squared residuals 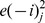
’s with PACo using **A**(-*i*);
    2. compute the jackknifed pseudovalues as *φ_ij_* = *n* · 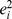 – (*n –*1) ·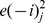.
  3. Set the jackknifed estimate 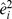 and its standard error *S_e_* as the arithmetic mean and standard error of the *φ_ij_*’s, respectively.
  4. Compute the approximate 95% confidence intervals of 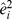 as *CI* = 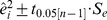.

This approach is illustrated in the application to the case studies below.

### Application to Case Studies

**Figure 2:**
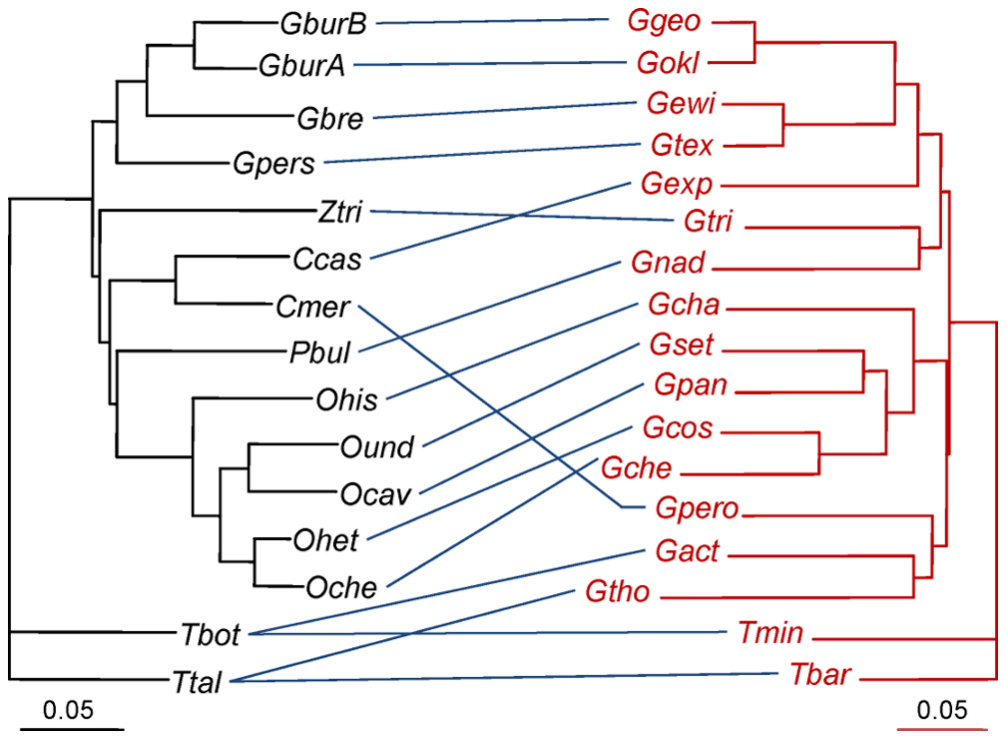
Phylogenetic trees of pocket gophers (left) and chewing lice (right). Blue lines represent host-parasite associations observed in nature. ***Gopher species abbreviations:*** Ccas: *Cratogeomys castanops*; Cmer: *C. merriami*; GburA: *Geomys bursarius halli*; GburB: *G. bursarius majusculus*; Gbre: *G. breviceps*; Gpers: *G. personatus*; Ocav: *Orthogeomys cavator*; Oche: *O. cherriei*; Ohet: *O. heterodus*; Ohis: *O. hispidus*; Ound: *O. underwoodii*; Pbul: *Pappogeomys bulleri*; Ztri: *Zygogeomys trichopus*; Tbot: *Thomomys bottae*; Ttal: *T. talpoides.* ***Louse species abbreviations:*** Gact: *Geomydoecus actuosi*; Gcha: *G. chapini*; Gche: *G. cherriei*; Gcos: *G. costaricensis*; Gewi; *G. ewingi*; Gexp: *G. expansus*; Ggeo: *G. geomydis*; Gnad: *G. nadleri*; Gokl: *G. oklahomensis*; Gpan: *G. panamensis*; Gpero: *G. perotensis*; Gset: *G. setzeri*; Gtex: *G. texanus*; Gtho: *G. thomomyus*; Gtri: *G. trichopi*; Tbar: *Thomomydoecus barbarae*; Tmin: *T.minor.*

We use data from two published studies to illustrate how PACo can be applied to real biological situations. The first one concerns the cophylogeny of pocket gophers and their chewing lice based on mitochondrial cytochrome oxidase I sequences [40] (Fig. 2). This model represents a classic example of host-parasite cospeciation [1], [39] that has been much used to test new methods in cophylogeny [7], [30], [34], [56]. The analyses were carried out with patristic and raw HKY85 [57] genetic distances, which were computed as explained in File S2.

**Figure 3:**
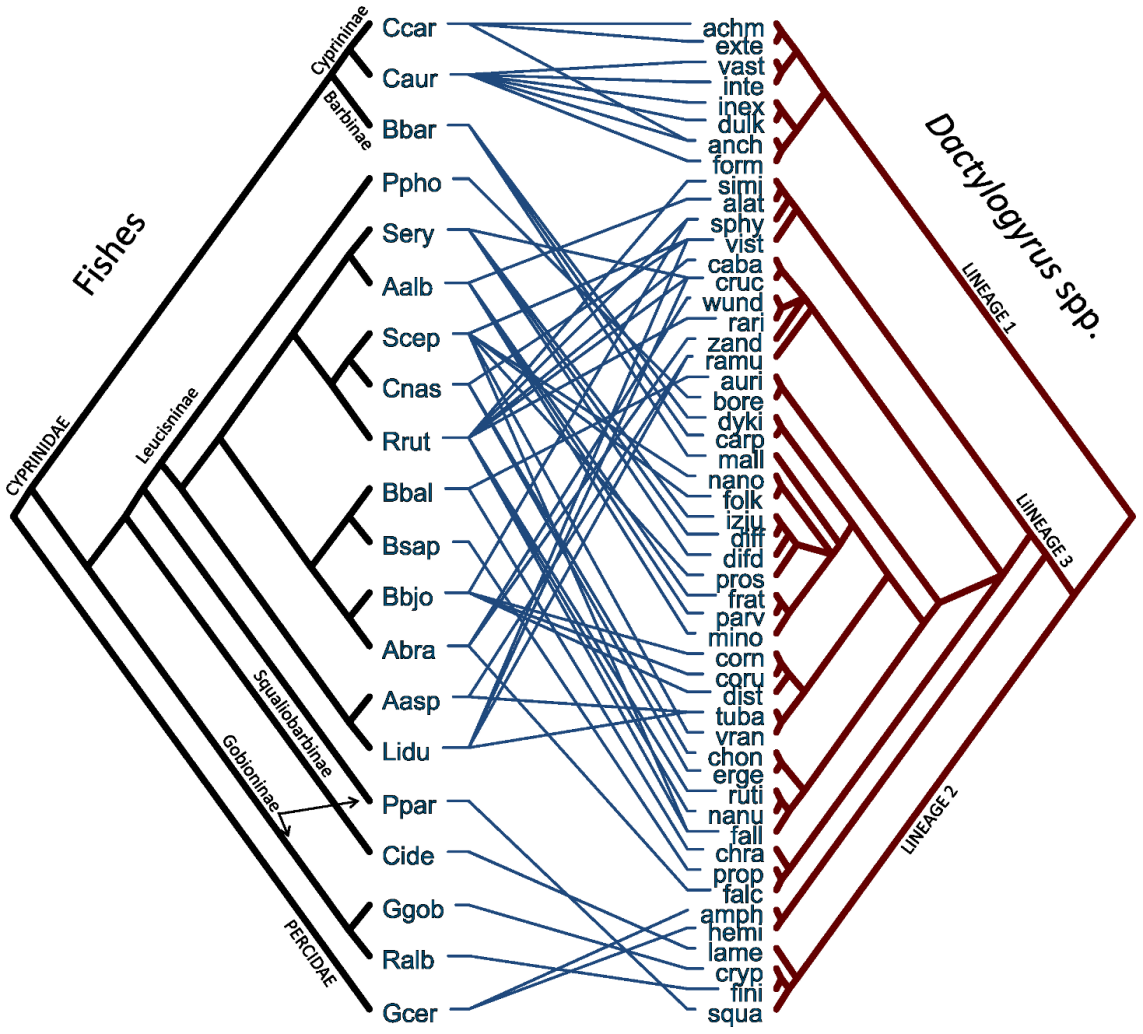
Tanglegram depicting the associations between 20 fishes and 51 *Dactylogyrus* spp (Monogenea). Lineages 1–3 of *Dactylogyrus* correspond to those recognized by Šimková et al. [32]. *Fish species abbreviations:* Aalb: *Alburnus alburnus*; Aasp: *Aspius aspius*; Abra: *Abramis brama*; Bbal: *Ballerus ballerus*; Bbar: *Barbus barbus*; Bbjo: *Blicca bjoerkna*; Bsap: *Ballerus sapa*; Caur: *Carassius auratus*; Ccar: *Cyprinus carpio*; Cide: *Ctenopharyngodon idella*; Cnas: *Chondrostoma nasus*; Gcer: *Gymnocephalus cernua*; Ggob: *Gobio gobio*; Lidu: *Leuciscus idus*; Ppar: *Pseudorasbora parva*; Ppho*: Phoxinus phoxinus*; Ralb: *Romanogobio albipinnatus*; Rrut: *Rutilus rutilus*; Scep: *Squalius cephalus*; Sery: *Scardinius erythrophthalmus*. *Dactylogyrus – specific-name abbreviations:* achm: *achmerovi*; alat: *alatus*; amph: *amphibothrium*; anch: *anchoratus*; auri: *auriculatus*; bore: *borealis*; caba: *caballeroi*; carp: *carpathicus*; chon: *chondrostomi*; chra: *chranilowi*; corn: *cornoides*; coru: *cornu*; cruc: *crucifer*; cryp: *cryptomeres*; difd: *difformoides*; diff: *difformis*; dist: *distinguendus*; dulk: *dulkeiti*; dyki: *dyki*; erge: *ergensi*; exte: *extensus*; falc: *falcatus*; fall: *fallax*; fini: *finitimus*; folk: *folkmanovae*; form: *formosus*; frat: *fraternus*; hemi*: hemiamphibothrium*; inex: *inexpectatus*; inte: *intermedius*; izju: *izjumovae*; lame: *lamellatus*; mall: *malleus*; mino: *minor*; nano: *nanoides*; nanu: *nanus*; parv: *parvus*; prop: *propinquus*; pros: *prostae*; ramu: *ramulosus*; rari: *rarissimus*; ruti: *rutili*; simi: *similis*; sphy: *sphyrna*; squa: *squameus*; tuba: *tuba*; vast: *vastator*; vist: *vistulae*; vran: *vranoviensis*; wund: *wunderi*; zand: *zandti*.

The second study involves 51 monogenean species of *Dactylogyrus* associated to 20 species of freshwater fishes [32]. This is clearly a more complex scenario with 60 host-parasite associations (Fig. 3), where the authors identified a relatively high number of intra-host parasite duplications together with some cospeciation and host-switching events [32]. We performed the analyses with phylogenetic patristic distances, which, for *Dactylogyrus* spp., were inferred from the published tree (Figure 2 in Šimková et al. [32]). For the fish species, in order to include *Romanogobio albipinnatus*, whose sequence was unavailable at the time of the original publication [32], we computed the patristic distances from a newly produced phylogeny based on cytochrome b sequences. (See the new phylogeny and details about its construction in File S2).

In both case studies, the trees and host-parasite associations were tested globally with PACo, ParaFit and HCT and the contribution of individual host-parasite links was evaluated by jackknifed estimates of the squared residuals (File S1) and ParaFitLink1 [7]. In the fish-*Dactylogyrus* model, the number of associations was too numerous to produce a clear global superimposition plot, but our emphasis was placed instead on the analysis of individual host-parasite links. ParaFitLink1 was carried out with CopyCat [24], which incorporates optimized algorithms for PCo and ParaFit to facilitate analyses with large datasets [23]. In order to obtain precise *P* values, all tests were performed with 100,000 permutations.

## Results

### Simulations

#### Type I error

The error rates for the 0.01 and 0.05 significance levels of the three tests are shown in Table 1. In one of the 32 simulations, PACo yielded a Type I error rate whose 95% confidence interval did not include the desired α value, whereas ParaFit failed under the same criterion in four instances and HTC produced correct Type I errors for all parameter combinations (Table 1). The results also suggest that ParaFit was slightly anti-conservative for the larger (20 host-20 parasite) phylogenies, as it tended to produce higher error rates than expected (Table 1). In practice, however, deviations from the expected values were small in the three tests and the plots of the empirical cumulative distribution functions (shown in File S2) indicated very close agreement to the expected uniform distribution for the full range of *P* values.

**Table 1:** Type I error estimates and their 95% confidence intervals for 0.01 and 0.05 significance levels.

	*α* = 0.01								
	PACo			ParaFit			HCT		
Simulations*	Est.	95% CI		Est.	95% CI		Est.		95% CI
10H 10P 10L	0.009	0.008–0.010		0.010	0.007–0.013		0.009		0.007–0.011
10H 10P 15L	0.009	0.007–0.011		0.010	0.008–0.013		0.008		0.006–0.010
10H 10P 20L	0.009	0.007–0.011		0.011	0.010–0.013		0.009		0.007–0.011
10H 10P 25L	0.009	0.008–0.011		0.012	0.009–0.014		0.008		0.007–0.011
10H 15P 10L	0.010	0.008–0.012		0.011	0.009–0.012		0.009		0.008–0.012
10H 15P 15L	0.010	0.008–0.012		0.010	0.009–0.013		0.009		0.007–0.011
10H 15P 20L	**0.008**	**0.006–0.009**		0.010	0.009–0.012		0.009		0.007–0.011
10H 15P 25L	0.009	0.008–0.012		0.011	0.008–0.013		0.009		0.008–0.012
15H 10P 10L	0.008	0.007–0.010		0.011	0.009–0.012		0.009		0.007–0.011
15H 10P 15L	0.009	0.008–0.011		0.011	0.009–0.013		0.010		0.008–0.012
15H 10P 20L	0.011	0.008–0.013		0.010	0.008–0.012		0.010		0.009–0.013
15H 10P 25L	0.010	0.009–0.012		0.011	0.009–0.013		0.011		0.009–0–014
20H 20P 20L	0.008	0.007–0.010		**0.013**	**0.011–0.015**		0.011		0.009–0.013
20H 20P 25L	0.010	0.008–0.014		0.012	0.010–0.014		0.009		0.008–0.012
20H 20P 30L	0.010	0.008–0.013		**0.012**	**0.011–0.014**		0.009		0.008–0.011
20H 20P 35L	0.010	0.008–0.013		0.011	0.009–0.013		0.011		0.009–0–014
	**α = 0.05**								
	**PACo**		**ParaFit**			**HCT**			
**Simulations** *	**Est.**	**95% CI**	**Est.**		**95% CI**	**Est.**		**95% CI**	
10H 10P 10L	0.052	0.049–0.057	0.049		0.046–0.054	0.048		0.043–0.052	
10H 10P 15L	0.047	0.044–0.052	0.051		0.047–0.056	0.046		0.042–0.051	
10H 10P 20L	0.047	0.044–0.052	0.050		0.046–0.055	0.048		0.045–0.052	
10H 10P 25L	0.052	0.047–0.057	0.051		0.046–0.055	0.052		0.046–0.056	
10H 15P 10L	0.048	0.045–0.052	0.052		0.047–0.057	0.049		0.045–0.054	
10H 15P 15L	0.050	0.047–0.054	0.054		0.049–0.059	0.052		0.048–0.057	
10H 15P 20L	0.050	0.045–0.055	0.049		0.044–0.053	0.050		0.046–0.055	
10H 15P 25L	0.051	0.046–0.055	0.051		0.046–0.055	0.050		0.045–0.054	
15H 10P 10L	0.047	0.043–0.053	0.048		0.045–0.054	0.048		0.044–0.053	
15H 10P 15L	0.048	0.043–0.053	0.049		0.045–0.054	0.047		0.043–0.052	
15H 10P 20L	0.053	0.049–0.057	0.053		0.049–0.057	0.054		0.049–0.058	
15H 10P 25L	0.050	0.047–0.055	0.050		0.046–0.054	0.051		0.047–0.055	
20H 20P 20L	0.047	0.043–0.051	**0.056**		**0.053**–**0.061**	0.052		0.047–0.055	
20H 20P 25L	0.050	0.045–0.055	**0.055**		**0.051**–**0.060**	0.051		0.047–0.055	
20H 20P 30L	0.052	0.048–0.057	0.054		0.050–0.058	0.051		0.048–0.055	
20H 20P 35L	0.050	0.047–0.054	0.052		0.048–0.057	0.050		0.046–0.055	

* Numbers indicate the number of hosts (H), parasites (P) and host-parasite links (L).

Type I errors were estimated with PACo (present study), Parafit [7] and HCT [34]. Est.: estimate; CI: confidence interval. Simulations where the 95% confidence interval did not include the desired α value are boldfaced.

#### Type II error

**Figure 4:**
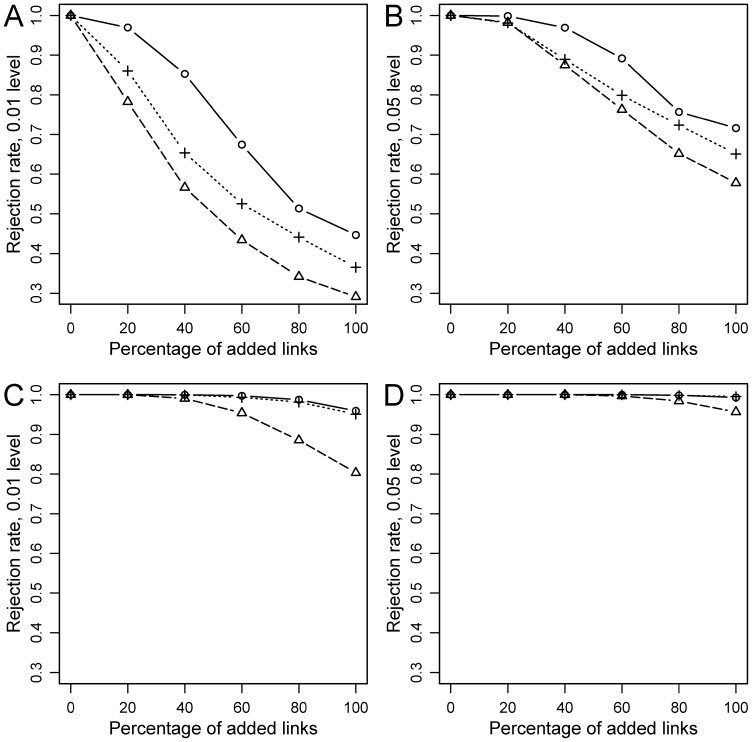
Statistical power for simulations under Approach 1 (Random links added). A, B: 10 host-10 parasite simulations; C, D: 20 host-20 parasite simulations. PACo (present study): circles (solid line); HCT [34]: crosses (dotted line); Parafit [7]: triangles (dashed line).

**Figure 5:**
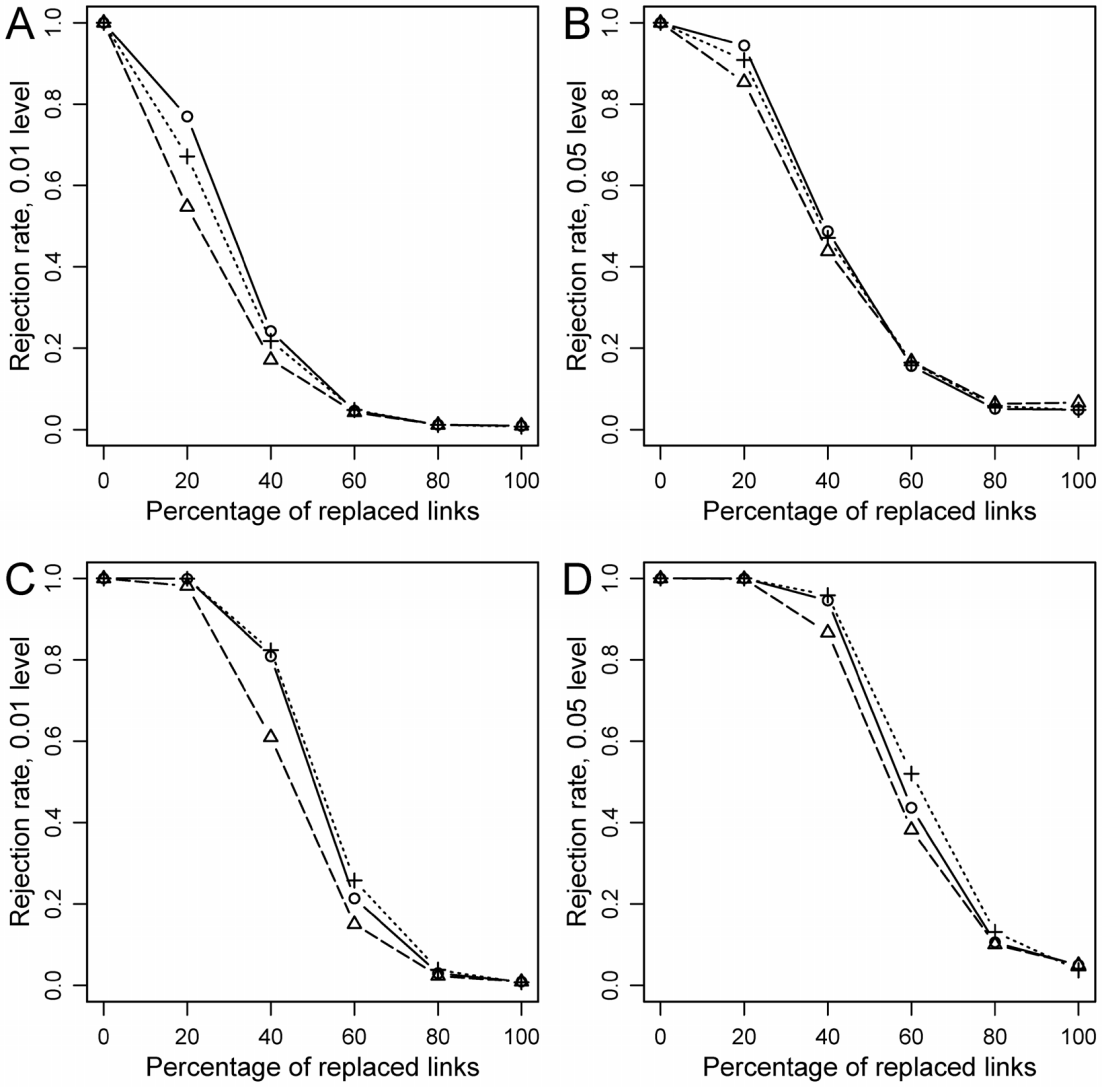
Statistical power for simulations under Approach 2 (Coevolutionary links replaced). A, B: 10 host-10 parasite simulations; C, D: 20 host-20 parasite simulations. PACo (present study): circles (solid line); HCT [34]: crosses (dotted line); Parafit [7]: triangles (dashed line).

**Figure 6:**
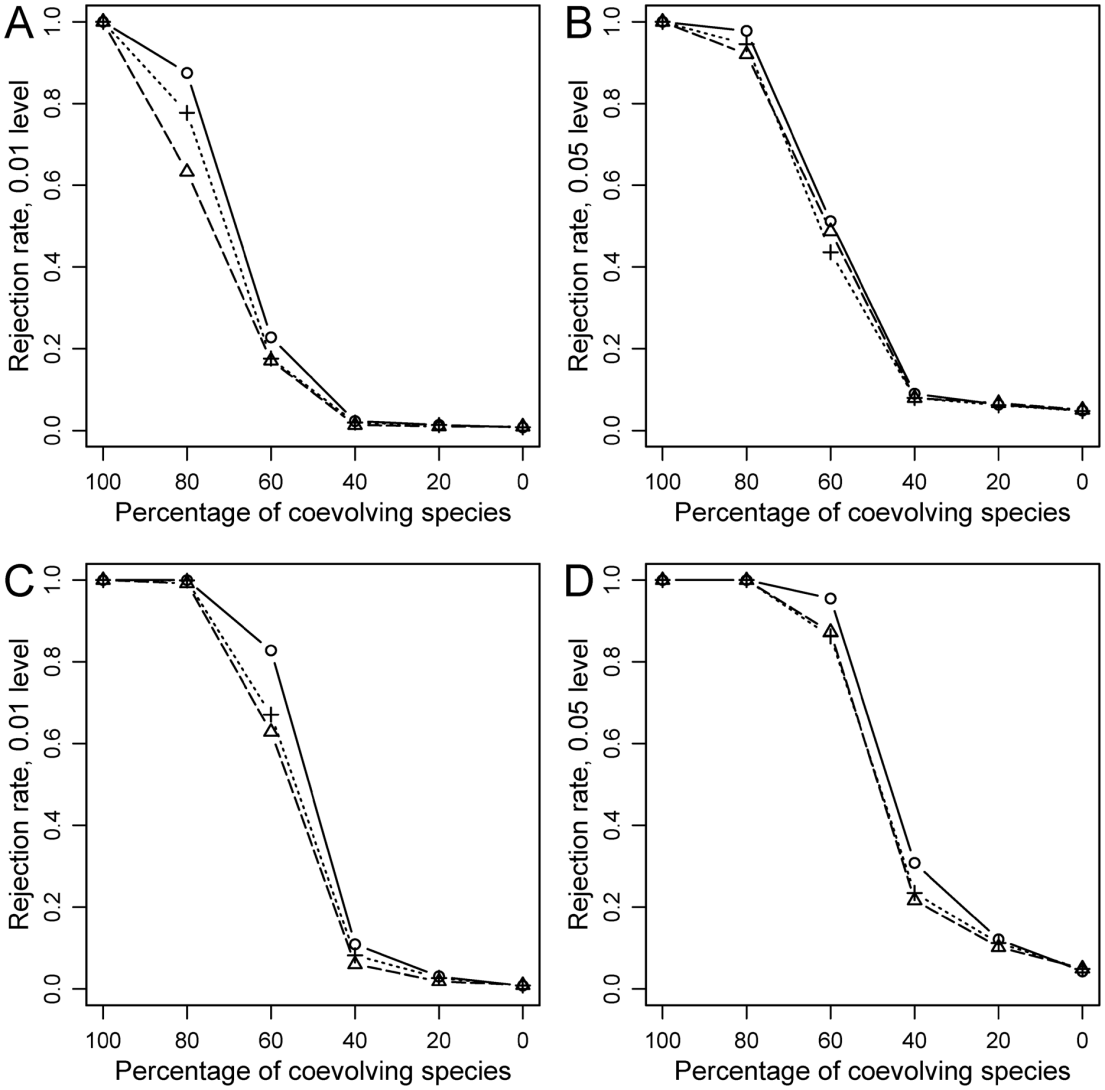
Statistical power for simulations under Approach 3 (Partly congruent trees). A, B: 10 host-10 parasite simulations; C, D: 20 host-20 parasite simulations. PACo (present study): circles (solid line); HCT [34]: crosses (dotted line); Parafit [7]: triangles (dashed line).

Three clear patterns, which could be generalized to the three tests, emerged. First, the rejection rate of *H_0_* was always 1 for the ideal coevolutionary setting (i.e., identical phylogenetic trees for hosts and parasites, and links at corresponding positions) and decreased as the amount of randomization increased (Figs. 4, 5, 6). Second, the reduction in power with level of randomization was more dramatic under simulations approaches 2 and 3, with respect to approach 1. This is probably so because the latter involves adding increasing random links to a perfect coevolutionary scenario. So, the coevolutionary signal diminishes as random links are added, but remains latent in the analyses. In contrast, in approaches 2 and 3 coevolutionary links are incrementally replaced with random counterparts. In fact, when all coevolutionary links were replaced by random ones, *H_0_* was made true and the rejection rates converged to the nominal *α* levels (either 0.01 or 0.05) (Figs. 5, 6). Third, for the same level of randomization, power was higher with larger (20 hosts-20 parasites) phylogenies in the three kinds of simulations (Figs. 4, 5, 6).

The main difference in performance among the three tests was observed under simulation approach 1, where PACo tended to show the highest power, followed by HCT and ParaFit (Fig. 4). So in a saturated coevolutionary model (a coevolutionary link relating every host-parasite pair), PACo seems less influenced by the effect of non-coevolutionary links than the other tests. Nevertheless, differences in power were less pronounced in the simulations with 20 hosts and 20 parasites (Fig. 4C, D) and indeed in this case the performance of the three tests was very similar at the 0.05 rejection level (Fig. 4D). Under simulation approaches 2 and 3, the three tests also behaved similarly, although PACo and HCT tended to show higher power than ParaFit, particularly at the 0.01 rejection level (Figs. 5, 6).

### Applications

#### Pocket gophers and chewing lice

The PACo analysis based on patristic distances yielded a 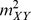 = 0.0731 with an associated permutational *P*<0.00001, which leads to rejection of *H_0_*. Likewise, the ParaFit global fit statistic was 0.0148 (*P* = 0.00002) and the HCT correlation was 0.4902 (*P* = 0.00004). Similar results were obtained using genetic distances: PACo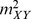 = 0.1159 (*P* = 0.00001); ParaFit global fit statistic = 0.0258 (*P* = 0.00039) and HCT correlation = 0.3978 (*P* = 0.00018). So the three methods indicate that it is very unlikely that the similarity between the diversification of pocket gophers and their lice has arisen by chance.

**Figure 7:**
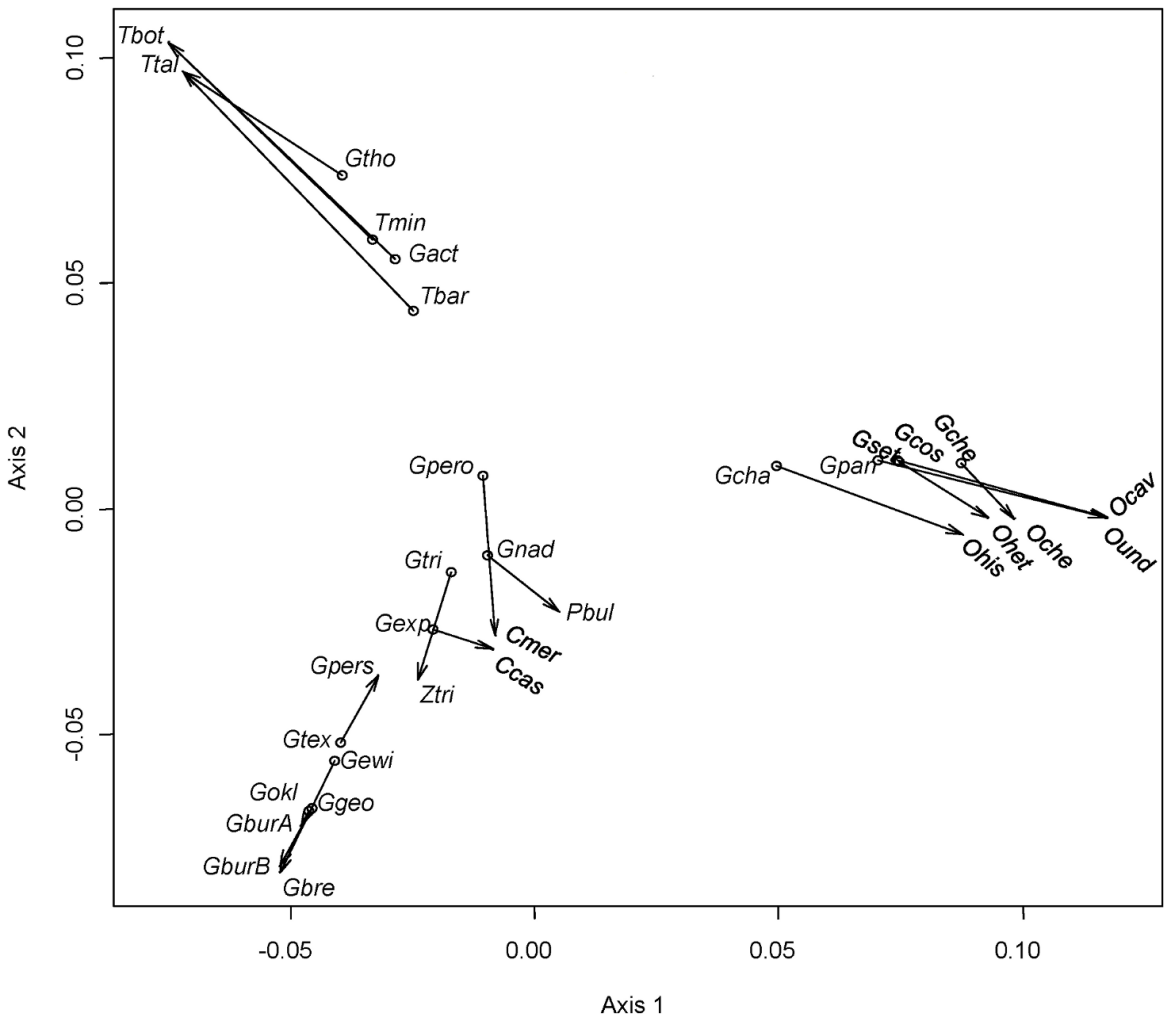
Procrustean superimpostion plot of pocket gophers and chewing lice. The ordinations of gopher and lice are Principal Correspondence Coordinates of patristic distances. The lice configuration (dots) has been rotated and scaled to fit the gopher ordination (arrow tips). Length of arrows represents the projection of residuals onto the first two axes. See Fig. 6 for species abbreviations.

**Figure 8:**
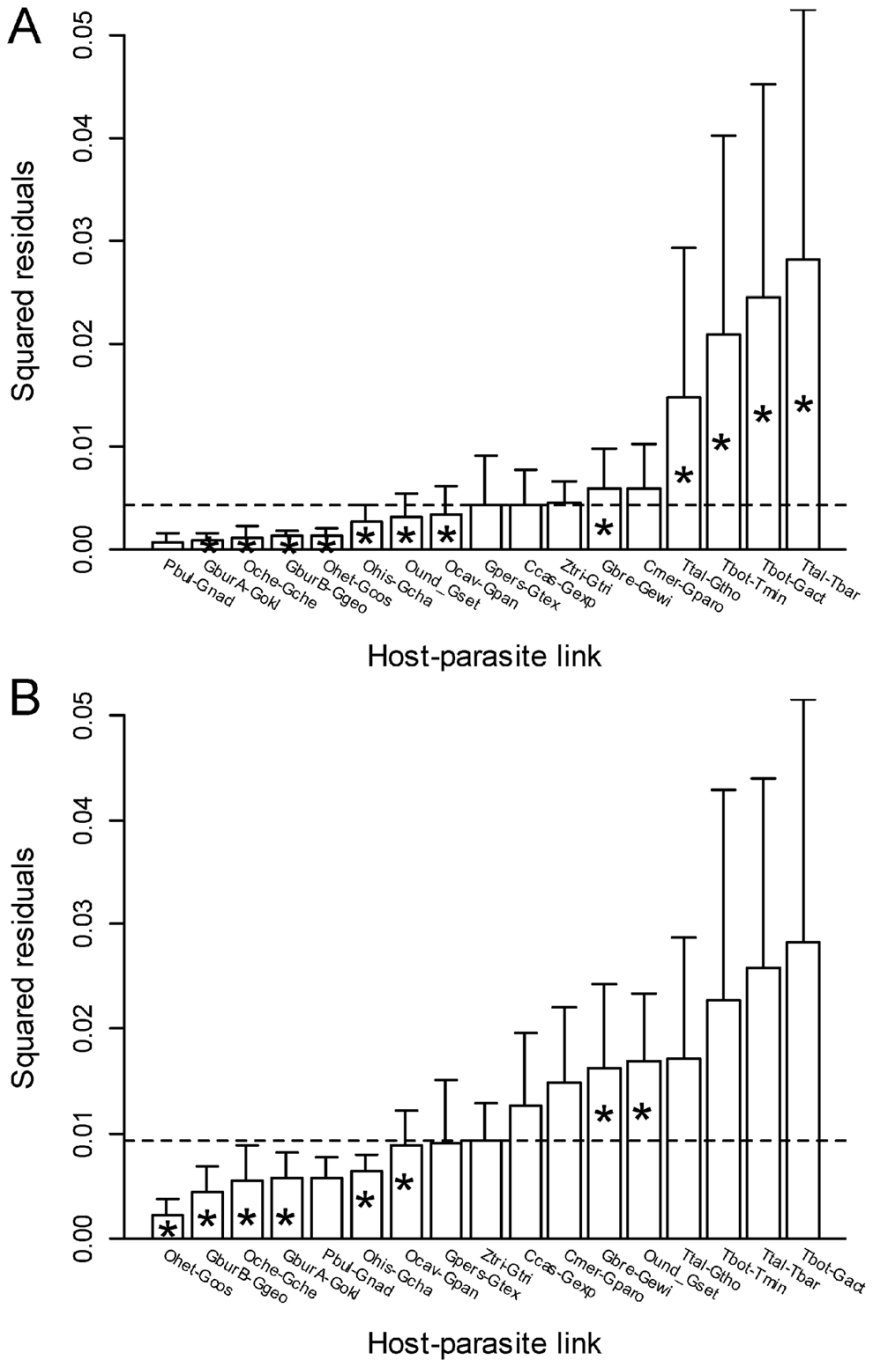
Pocket gophers and chewing lice: contributions of individual host-parasite links to the Procrustean fit. Jacknifed squared residuals (bars) and upper 95% confidence intervals (error bars) resulting from applying PACo to (A) patristic and (B) genetic distances. Asterisks identify links significantly supported (*α* <0.05) by ParaFitLink1 [7]. To ease comparisons the median squared residual value is shown (dashed line). See Fig. 2 for species abbreviations.

The agreement of the fit between the gopher and louse phylogenies can be visualised with a Procrustes superimposition plot onto the first two axes. The plot corresponding to patristic distances (Fig. 7) suggests four groups of host-parasite associations: One is formed by *Orthogeomys* spp. and their associated louse species, whose phylogeny closely mirror that of their hosts. A second group concerns *Geomys* spp. and species of *Geomydoecus*, which is topologically close to a third group, formed by species of *Pappogeomys*, *Cratogeomys* and *Zygogeomys* and their associated lice. The fourth group consists of the two species of *Thomomys* associated to four lice species. A similar grouping pattern was obtained with the HKY85 genetic distances (Fig. S1 in File S1). The host-parasite links in Figure 7 are represented by arrows whose length roughly represents the corresponding residuals. However, these distances in the two-dimensional plot underestimate the actual residuals in a full-dimensional space and caution should be exercised when evaluating residuals in this manner. For instance, the superimposition plot (Fig. 7) would suggest that the residual *Cratogeomys castanops* – *G. expansus* is smaller than the residual *Orthogeomys hispidus* –*Geomydoecus chapini*, when it is actually the opposite (Fig. 8).

The bar plots of squared residuals, using both patristic and genetic distances (Fig. 8), indicate that most links associated to the gopher species of genera *Orthogeomys* and *Geomys* contribute relatively little to
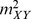
and thus likely represent coevolutionary links. In general and although not entirely coincidental, links with low squared residuals tended to be identified as coevolutionary with the ParaFitLink1 test, but the opposite did not apply (at least for the analysis involving patristic distances, Fig. 8A). However, as noted above, setting the *α* level of ParaFitLink1 to 0.05 results in an anti-conservative test and some of the host-parasite links marked as significant may not represent actual coevolutionary associations. In fact, conflicting evidence from ParaFitLink1 applied to patristic and genetic distances was obtained. The links related to the species of *Thomomys* were considered as coevolutionary in the former but not in the latter type of analyses (Fig. 8). These links were associated to the highest residuals, but the jackknife estimation revealed their broad confidence intervals indicating uncertainty about their actual values. Results of our residual analyses with patristic and raw distances were more congruent, although some differences concerning the status of the *O. underwoodi – G. setseri* link were also observed.

**Fish and** ***Dactylogyrus*** **spp.** Šimková et al. [32] identified three lineages of *Dactylogyrus* (Lineages 1–3) that were associated respectively in our tanglegram to Cyprininae, Gobioninae-Squaliobarbinae-Percidae, and mostly Leucisninae (Fig. 3). The three global-fit methods provided clear support for this overall congruence (PACo 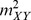 = 13.29, *P*<0.00001; ParaFit global statistic = 4.12, *P*<0.00005; HCT *r* = 0.505, *P*<0.00001).

**Figure 9:**
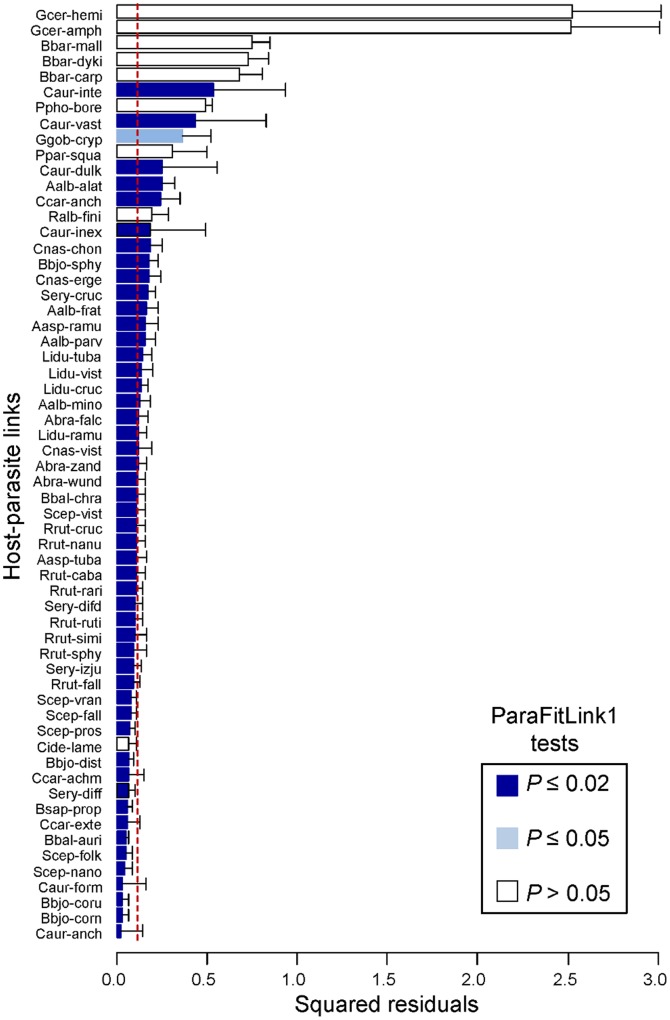
Fish and *Dactylogyrus* spp.: contributions of individual host-parasite links to the Procrustean fit. Jacknifed squared residuals (bars) and upper 95% confidence intervals (error bars) resulting from applying PACo to patristic distances. Results of the ParaFitLink1 analysis [7] for each link are indicated by the bar colour. To ease comparisons the median squared residual value is shown (red dashed line). See Fig. 3 for species abbreviations.

Both ParaFitLink 1 and PACo identified links that were clearly incongruent with a coevolutionary history. *Barbus barbus* and *Gymnocephalus cernua* apparently acquired their parasites from host-switches of species associated to the Leucisninae (Figs. 3, 9). The ParaFitLink1 analysis considered 50 of the 60 host-parasite links as coevolutionary at the default 0.02 significance level of CopyCat (Fig. 9). As in the preceding example, our evidence points to the anti-conservative nature of this test, because a large number of significant links included associations of fishes, e.g. *Rutilus rutilus* or *Leuciscus idus*, with paraphyletic groups of parasites. Although our residual approach did not show enough resolution to solve all these conflicting relationships, it could at least provide insight into the nature of some of them. In the Cyprininae-Lineage 1 associations, for instance, all residuals associated to links of *Carassius auratus* with the *Dactylogyrus inexpectatus* – *D. formosus* clade were smaller (and their confidence intervals contained zero) than those with *D. vastator* and *D. intermedius*. Likewise, the links of *Cyprinus carpio* with *D. achmerovi* – *D. extensus* had smaller residuals than the link between the former and *D. anchoratus*. This suggests two coevolutionary associations between *C. auratus* and the *D. inexpectatus* – *D. formosus* ancestor and between *C. carpio* and the *D. achmerovi* – *D. extensus* ancestor (followed by intrahost duplications), whereas the rest of the links would represent host-switches within the Cyprininae (Figs. 3, 9). Similarly, while ParaFitLink1 was inconclusive about the host associations of Lineage 2 (none of them were significant at the 0.02 level, Fig. 9), PACo indicated a possible coevolutionary relationship with the Squaliobarbinae, given the low squared residual associated to the *Ctenopharyngodon idella* – *D. lamellatus* link (Fig. 9). However, further work is needed because the Squaliobarbinae and Gobioninae clades were poorly supported in both Šimková et al. [32] and our phylogram (File S2).

## Discussion

We have demonstrated an application of Procustes analysis to cophylogeny. Procrustes fitting is a well-established method in morphometrics [58] but its potential range of applications in other biological areas is very wide. For instance, PROTEST, a Procrustes variant designed for comparison between ecological matrices [45], has paved the way for its use in community ecology (e.g., [59], [60], [61], [62], [63]). In contrast, the use of Procrustean approaches in phylogenetic contexts has been rather modest. Applications include the analysis of microarray data [64] and phylogeography for characterization of genetic structure in geographical space [65], [66]. In addition, Choi and Gomez [29] presented a method for comparison of phylogenetic trees which is similar to PACo in using Procrustean superimposition of PCo configurations. However, the method differs in that it did not contemplate multiple associations between the leaves. We believe that Procrustean fitting has much to offer to cophylogenetic analysis, due to its high versatility. For instance, Schardl et al. [35] developed an efficient algorithm using ultrametric trees for study of codivergence between hosts and parasites that could be readily adapted to a Procrustean approach. In the same vein, Nieberding et al. [37] proposed a ParaFit-based method to study the influence of ecological traits and their geographic variation in explaining congruence between host and parasite taxa. Given the commonalities between PACo and ParaFit, it seems plausible to develop a Procrustean tool with the same purpose.

PACo shares the advantages of ParaFit and HCT of not requiring fully resolved phylogenies and allowing multiple host-parasite associations. These tests can be carried out with any distance metric, such as raw genetic or patristic distances, as illustrated herein with the gopher-lice example. Whereas raw genetic distances represent the number of substitutions differences between two species (or sequences), patristic distances measure the amount of genetic divergence accounting for the divergence time between species (or populations) [67] and thus contain more implicit evolutionary information. However, patristic distances can artificially bring closer species that have small branch lengths and separate species with longer branches [68]. Having this in mind, comparing results given by different types of distances may produce better insight into the cophylogentic process under study. Additionally, PACo could be used with other metrics, such as phenetic distances, to study, for instance, the coevolution of a parasite-trait on different hosts. However, this falls outside the scope of the present study.

We acknowledge that distance-based methods are not the only way (and not necessarily even the best way) to analyse cophylogenetic patterns. It can be argued that distance-based approaches actually test for congruence between matrices of evolutionary distances in lieu of strict topological congruence between the trees. PACo is applied to the resulting Euclidean configurations, which are more remote from the true tree than an estimated phylogeny would be, and thus can be considered as a more noisy representation. In fact, tree space has a much lower dimension that Euclidean space [27] and, consequently, pairwise Euclidean distances may not accurately represent tree topologies. However, PCo decomposition of phylogenetic distance matrices seems to produce a reasonable representation of the phylogenetic tree [7], [29], [42], [68]. The fact that PCo coordinates are not in the same space as the trees does not invalidate their use for testing cophylogenetic patterns, but represent a limitation of these tests. One strategy to alleviate this problem could be to consider the position of nodes in the trees to avoid biases in sampling of pairwise distances, as shown with ultrametric trees [35]. It would be worth determining whether this approach can be generalized to additive trees, but, in any case, the spatial properties of cophylogenetic trees remain largely unexplored [69] and therefore much further work in this area is clearly needed.

Likewise, future studies would need to explore technical refinements for enhanced performance of PACo, particularly in evaluating the effects of individual links or groups of links on the global fit. As in ParaFit, our test relies in the ability of transforming phylogenies (or distances matrices) into PCo ordinations. The use of non-Euclidean distances (as usually happens with phylogenetic data) leads to negative eigenvalues and distortions of the relationships among the data points [49]. To tackle this problem, we applied the Cailliez correction for negative eigenvalues, which is commonly used in this situation [38], [44]. De Vienne et al. [42] recently proposed a new, more efficient, correction based on computing the element-wise square root of the patristic distances that deserves attention in future studies. In the same vein, PACo is based on least-squares fitting, which is the method used by most software, but is known to be relatively vulnerable to outliers [70]. Resistant-fit techniques that potentially produce more robust solutions by down-weighting the influence of unusual points have been proposed [71], [72]. This approach has proved useful to detect local regions of similarity between phylogenetic trees and to identify outliers relative to a common shared structure [29]. Other studies have considered Procrustes fitting under Bayesian frameworks [72], [73].

Despite these open issues, PACo includes several innovative elements with respect to ParaFit and HCT that can make it attractive to potential users. First, PACo is unique in that it produces an informative graphical output for both global evaluation of the fit and assessing the contribution of the individual host-parasite links. The application to the pocket gopher – chewing louse model revealed, for instance, the distinctness of the relationship between *Orthogeomys* spp. and their associated lice, where cladogenesis of the hosts was mirrored by that of their parasites (Fig. 6). We also showed that the graphical representation of squared residuals is a reasonable alternative to the ParaLink1 test, enabling more elaborate validations as particularly shown in the fish-*Dactylogyrus* example. Second, PACo is a more specific test than ParaFit and HCT. Whereas ParaFit and HCT analyse correlation between phylogenies of the associated taxa, PACo is especially suited for systems where dependence of one phylogeny upon another is assumed. Thus it is ideal to test for the common coevolutionary model that assumes that parasites that spend part of all their life in or on their hosts track the phylogeny of their hosts [2], [39]. In other situations, parasites have been proposed as potential determinants of host speciation [74], [75] and consequently PACo could readily accommodate to this scenario by fitting the host phylogeny onto the parasite phylogeny. Likewise, given that historical area relationships are expected to determine taxa diversification but not the opposite, our method is more suitable than ParaFit and HCT to evaluate diversification of taxa in biogeographical settings. Third, our method is statistically reliable as shown by its very good performance in terms of Type I and Type II errors. The simulations indicated superior Type I error performance than ParaFit for the largest phylogenies (20 hosts and 20 parasites) tested. In addition, PACo stands out by its overall higher statistical power, particularly, for saturated coevolutionary host-parasite scenarios. For greater usability, PACo can be implemented in the public-domain statistical software R (File S1) in a reasonable amount of computing time, which affords the analysis of large datasets. In conclusion, PACo is a new tool that benefits from the versatility of Procustes fitting to provide a simple and intuitive way to test statistically phylogenetic congruence between phylogenetic trees, and phylogenetic distance matrices in general, of associated taxa.

## Supporting Information

File S1 **PACo in R – User Guide.** Annotated R code to carry out all the analyses described in the present paper is provided. Its use is demonstrated with the phylogenies of pocket gophers and their chewing lice.
(PDF)

File S2 **Methodological details and additional results.** This file includes details about the phylogenetic methods used and plots of empirical cumulative distribution function of the *P* values obtained in simulations, showing the correctness of the Type I errors of the tests compared for any for any significance level.
(PDF)
