# Supplementary material for: PACo: A Novel Procrustes Application to Cophylogenetic Analysis
Source: PLoS One. 2013 Apr 8;8(4):e61048. doi: 10.1371/journal.pone.0061048 (PMC3620278; doi:10.1371/journal.pone.0061048)
Supplement: File S1 — PACo in R – User Guide. Annotated R code to carry out all the analyses described in the present paper is provided. Its use is demonstrated with the phylogenies of pocket gophers and their chewing lice. (PDF) [file pone.0061048.s001.pdf]

File S1 – Supporting information for

PACo: a novel Procrustes application to cophylogenetic analysis

PACo in R – User Guide

Juan Antonio Balbuena[1]*, Raúl Míguez-Lozano[1], Isabel Blasco-Costa[2]

[1] Cavanilles Institute of Biodiversity and Evolutionary Biology, University of Valencia, Valencia, Spain.

[2] Institute of Parasitology, Academy of Sciences of the Czech Republic, České Budějovice, Czech Republic.

*E-mail: j.a.balbuena@uv.es

Introduction

We present an R [1] script to carry out PACo (**P**rocrustes **A**pproach to **Co**phylogeny), an application of Procrustes analysis for comparison of phylogenetic trees of associated organisms, such as hosts and parasites. PACo provides a residual sum of squares of the Procrustean fit that measures the congruence between two given phylogenies and uses a permutation approach to test its significance. The analysis allows for multiple host-parasite associations and different number of hosts and parasites. Because in the Procrustean superimposition the host matrix is kept fixed, whereas the parasite matrix is rotated and scaled to fit the former, PACo tests the classical view of whether the parasite phylogeny is constrained by the host phylogeny [2]. This implies that the null hypothesis tested is slightly different from that of previous tests of phylogenetic congruence [3-5]. In addition to hypothesis testing, PACo provides a superimposition plot enabling a graphical comparison of the fit of the host-parasite associations, and a residual bar chart for evaluation of the contribution of the individual host-parasite associations to the global fit.

The R script

All the computations described in the accompanying paper can be carried out with the R script below. R runs on a wide variety of Linux/Unix platforms, Windows and MacOS and can be downloaded at http://www.r-project.org/. In addition to the basic R install, two dedicated packages need to be installed to implement PACo: `ape` [6], required for handling of phylogenetic data and Principal Coordinates Ordination and `vegan` [7], required for Procrustes fitting. (See http://cran.r-project.org/doc/manuals/R-admin.html#Installing-packages for details on how to install R packages). In order to assist users with little or no experience with R, we provide annotations to the script. The analyses can be implemented by cutting and pasting the code below in an opened R console. The text in red identifies parameters that can be customized to adapt the analysis to the user's needs.

The script is demonstrated with the phylogenies of pocket gophers and their chewing lice based on the mitochondrial cytochrome oxidase I sequences of Hafner et al. [8], which represents a classical example of host-parasite cospeciation [9]. The input files required are shown in the Appendix below and can be downloaded, together with a fully annotated R script, at http://www.uv.es/cophylpaco/index.html.

First, load the two packages required. At the R prompt, write

```
library(ape)

library(vegan)
```

Data input

Input files should be in plain text format either space- or tab-delimited. (See Appendix for examples). Three files are required. Two of them encapsulate the host and parasite phylogenies, respectively, and will eventually be transformed into distance matrices between host or parasite taxa. The third one consists of a binary matrix coding the host-parasite associations, where host species are arranged in rows and parasites in columns; 1's indicate occurrence of a given parasite in a given host and 0's denote parasite absences in the host. The input files should **include taxa labels that have to match exactly in the three files**. Any mismatch will cause execution errors and/or incorrect results. (For clarity of the graphical output, short labels are recommended.)

The following syntax reads the matrix of host-parasite associations and computes the number of associations (`NLinks`) required for further computations:

```
HP <- as.matrix(read.table("PACo/example/gophers/g-l_links.txt", header=TRUE))

NLinks = sum(HP)
```

As illustrated by the foregoing code, the path pointing to the file system location should be indicated in quotation marks. [Windows users should also note that folder names are separated by forward (/), instead of backward (\) slashes.] Alternatively, one can open the input files interactively with `file.choose()`, e.g.

```
HP <- as.matrix(read.table(file.choose(), header=TRUE))
```

The syntax to load the phylogenetic input depends on the type of data used. There are three possibilities: (a) phylogenetic trees, (b) aligned sequences or (c) distance matrices.

a) Phylogenetic trees

Use the `read.tree` or `read.nexus` functions to open tree files in Newick or Nexus formats, respectively. For instance,

```
TreeH <- read.tree("PACo/example/gophers/phylo_gopher.tre")

TreeP <- read.tree("PACo/examp le/gophers/phylo_lice.tre")
```

The host and parasite trees are then transformed for further analysis into respective matrices of patristic distances (host.D and para.D):

```
host.D <- cophenetic(TreeH)

para.D <- cophenetic(TreeP)
```

b) Aligned sequences

The `read.dna` function can read sequences in different formats. (Consult the `ape` package tutorial for details). In this example the files are in fasta format:

```
seqH <- read.dna("PACo/example/gophers/alig_gopher.fasta",format="fasta")

seqP <- read.dna("PACo/example/gophers/alig_lice.fasta",format="fasta")
```

Next, the sequence data is used to compute genetic distance matrices of hosts and parasites:

```
host.D <- dist.dna(seqH, model = "F84", as.matrix=TRUE)

para.D <- dist.dna(seqP, model = "F84", as.matrix=TRUE)
```

The `dist.dna` function allows choosing among a range of evolutionary models [6]. The example above uses the Felsenstein's substitution model (F84) [10].

c) Distance matrices

Text files with square distance or dissimilarity matrices can also be used as input:

```
host.D <- as.matrix(read.table("PACo/example/gophers/dist_gopher.txt",

header=TRUE))

para.D <- as.matrix(read.table("PACo/example/gophers/dist_lice.txt",

header=TRUE))
```

The files dist_gopher.txt and dist_lice.txt (see Appendix), contain genetic distances computed with PAUP* [11] using the HKY85 [12] substitution model.

All the demonstrations below use this third input option (HKY85 genetic distances).

Procustean Superimposition

The host and parasite distance matrices are first sorted according, respectively, to the order of rows (hosts) and columns (parasites) of the host-parasite association matrix:

```
host.D <- host.D[rownames(HP),rownames(HP)]

para.D <- para.D[colnames(HP),colnames(HP)]
```

The `PACo` function, defined below, transforms the host and parasite distance matrices into the respective matrices of Principal Coordinates (`pcoa` of `ape`) and duplicates taxa (if necessary) to accommodate multiple host-parasite associations:

```
PACo <- function (H.dist, P.dist, HP.bin)

{HP.bin <- which(HP.bin > 0, arr.in=TRUE)

H.PCo <- pcoa(H.dist, correction="cailliez")$vectors
```

```
P.PCo <- pcoa(P.dist, correction="cailliez")$vectors

H.PCo <- H.PCo[HP.bin[,1],]

P.PCo <- P.PCo[HP.bin[,2],]

list (H.PCo = H.PCo, P.PCo = P.PCo)}
```

`PACo` is then applied to the input and a Procrustes fit is carried out:

```
PACo.fit <- PACo(host.D, para.D, HP)

HP.proc <- procrustes(PACo.fit$H.PCo, PACo.fit$P.PCo)
```

The program produces the following output:

```
Warning message:
In procrustes(PACo.fit$H.PCo, PACo.fit$P.PCo) :
  X has fewer axes than Y: X adjusted to comform Y.
```

This indicates that the host input matrix has fewer columns than the parasite counterpart. No action by the user is required since the narrower matrix is completed with zero columns [13].

To visualize the host-parasite superimposition plot, as show in Figure S1.1:

```
HostX <- HP.proc$X

ParY <- HP.proc$Yrot

plot(HostX, asp=1, pch=46)

points(ParY, pch=1)

arrows(ParY[,1], ParY[,2], HostX[,1], HostX[,2], length=0.12, angle=15,

xpd=FALSE)

HostX <- unique(HP.proc$X)

ParY <- unique(HP.proc$Yrot)

identify(ParY[,1], ParY[,2], rownames(ParY), offset=0.3, xpd=FALSE, cex=0.8)

identify(HostX[,1], HostX[,2], rownames(HostX),offset=0.3, xpd=TRUE, cex= 0.8)
```

With `identify` the user can label the data points interactively by clicking near each data point on the plot. First label the parasite locations and then those of the hosts.

Goodness-of-fit test

The following code computes the residual sum of squares m^2_{XY} and performs a randomization of the host-parasite association matrix to establish the probability *P* under H_0:

```
m2.obs <- HP.proc$ss

N.perm = 100000
```

where `N.perm` sets the number of random permutations of the host-parasite association matrix. For high precision of the *P* estimate, 100,000 permutations were used in the accompanying paper. Although computing time was not prohibitive (some 24 min. on a PC equipped with an Intel Core 2 CPU 6600 @ 2.40GHz processor), in most situations ≤ 10,000 permutations would be sufficient for hypothesis testing.

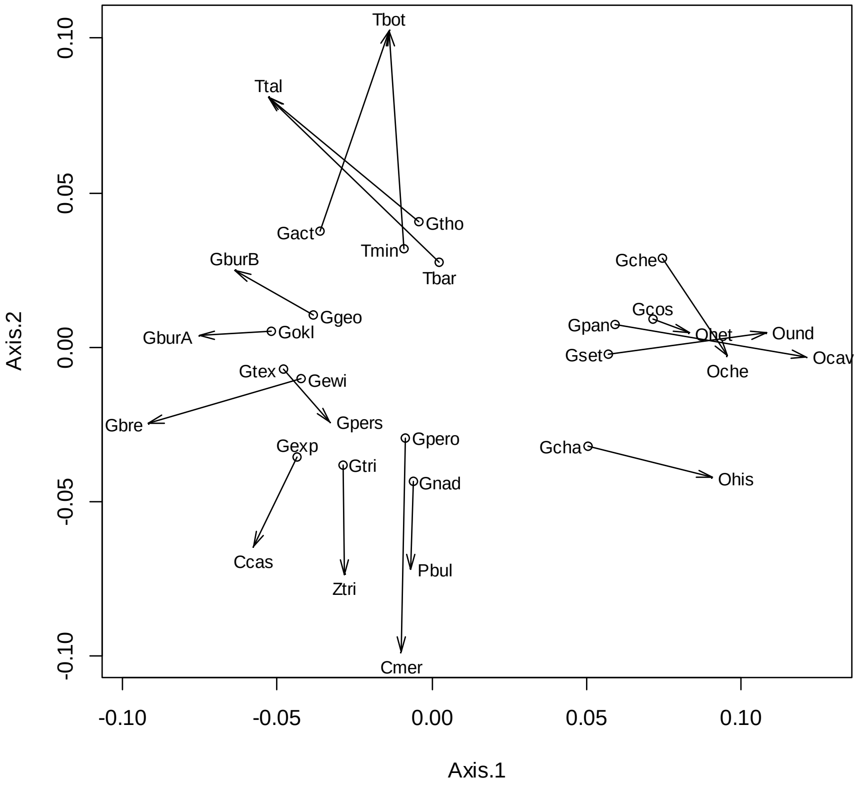

Figure S1. Procrustean superimposition plot of pocket gophers and chewing lice. The ordinations of gopher and lice are Principal Correspondence Coordinates of HKY85genetic distances. The lice configuration (dots) has been rotated and scaled to fit the gopher ordination (arrow tips). See Appendix for abbreviations of taxa.

```
P.value = 0

set.seed(.Random.seed[trunc(runif(1,1,626))])
```

The last line sets the randomization seed. To obtain reproducible sets of permutations, use instead an integer of choice, for instance `set.seed(5)`.

```
for (n in c(1:N.perm))

{if (NLinks <= nrow(HP) | NLinks <= ncol(HP))

      {flag2 <- TRUE

            while (flag2 == TRUE)

            {HP.perm <- t(apply(HP,1,sample))

            if(any(colSums(HP.perm) == NLinks)) flag2 <- TRUE

            else flag2 <- FALSE}
```

```
      } else { HP.perm <- t(apply(HP,1,sample))}
      PACo.perm <- PACo(host.D, para.D, HP.perm)
      m2.perm <- procrustes(PACo.perm$H.PCo, PACo.perm$P.PCo)$ss
      write(m2.perm, file="PACo/example/m2_perm.txt", sep="\t", append=TRUE)
      if (m2.perm <= m2.obs){P.value = P.value + 1}
}
```

Although the `write` function above is not essential for the analysis (and can be omitted), it is useful if one wishes to save the set of residual sum of squares generated at each permutation for further reference. Note the path pointing to the location where the file will be saved. Given that `append=TRUE`, the file created (m2_perm.txt) should be deleted or renamed prior to a new analysis. Otherwise the values generated in the new run will be appended to those produced in the previous one.

To conclude the goodness-of-fit test:

```
P.value <- P.value/N.perm
cat(" The observed m2 is ", m2.obs, "\n", P.value, " based on ", N.perm,"
permutations.")
```

The following output is produced:

```
      There were 50 or more warnings (use warnings() to see the first 50)
      The observed m2 is  0.1158733
       P-value =  1e-05 based on  1e+05  permutations.
```

(The warnings are originated by each of the Procrustes analyses with the permuted host-parasite association matrix and result from the different number of columns in the host and parasite matrices. As noted above this has no effect on the analysis).

So m^2_{XY} = 0.1159. In only one of the 100,000 random permutations the residual sum of squares was smaller than this value (i.e., $P = 10^{-5}$) and congruence between the host and parasite phylogenies is statistically significant at the conventional significance level of 0.05.

Evaluation of host-parasite links

As justified in the accompanying article, the contribution of each host-parasite to the global fit can be assessed with a jackknife procedure that estimates the squared residual and its 95% confidence interval of each individual link:

```
HP.ones <- which(HP > 0, arr.in=TRUE)
SQres.jackn <- matrix(rep(NA, NLinks**2), NLinks)
```

```
      colnames (SQres.jackn) <- paste(rownames(HostX),rownames(ParY), sep="-")
t.critical = qt(0.975,NLinks-1)
for(i in c(1:NLinks))
{HP.ind <- HP
HP.ind[HP.ones[i,1],HP.ones[i,2]]=0
PACo.ind <- PACo(host.D, para.D, HP.ind)
Proc.ind <- procrustes(PACo.ind$H.PCo, PACo.ind$P.PCo)
res.Proc.ind <- c(residuals(Proc.ind))
res.Proc.ind <- append (res.Proc.ind, NA, after= i-1)
SQres.jackn [i, ] <- res.Proc.ind}
SQres.jackn <- SQres.jackn**2
SQres <- (residuals (HP.proc)**2
SQres.jackn <- SQres.jackn*(-(NLinks-1))
SQres <- SQres*NLinks
SQres.jackn <- t(apply(SQres.jackn, 1, "+", SQres))
phi.mean <- apply(SQres.jackn, 2, mean, na.rm = TRUE)
phi.UCI <- apply(SQres.jackn, 2, sd, na.rm = TRUE)
phi.UCI <- phi.mean + t.critical * phi.UCI/sqrt(NLinks)
pat.bar <- barplot(phi.mean, names.arg = " ", space = 0.25, col="white", xlab=
"Host-parasite link", ylab= "Squared residuals", ylim=c(0, max(phi.UCI)),
cex.lab=1.2)
text(pat.bar, par("usr")[3] - 0.001, srt = 330, adj = 0, labels =
colnames(SQres.jackn), xpd = TRUE, font = 1, cex=0.6)
arrows(pat.bar, phi.mean, pat.bar, phi.UCI, length= 0.05, angle=90)
abline(a=median(phi.mean), b=0, lty=2)
```

The foregoing code produces a bar chart of squared residuals (Fig. S1.2). Most links related to gopher species of *Orthogeomys*, *Geomys* and *Pappogeomys* contributed relatively little to m_{XY}^2 and thus likely represent coevolutionary links. The links related to *Thomomys* spp. showed the highest residuals but their confidence intervals were quite broad (Fig. S1.2). Thus, it is difficult to evaluate their contribution to the cophylogenetic pattern observed.

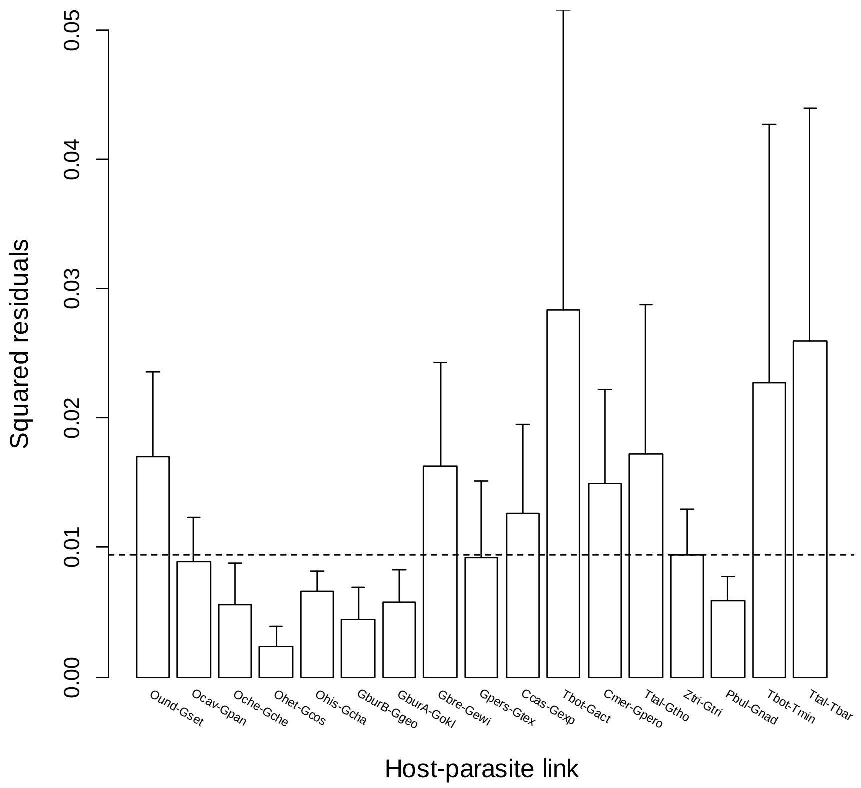

Figure S2. Jacknifed squared residuals (bars) and upper 95% confidence intervals (error bars) associated to each gopher-louse link. PACo was applied to HKY85 genetic distances. The dashed line indicates the median squared residual value. See Appendix for abbreviations of taxa.

References

1. R Development Core Team (2011) R: A language and environment for statistical computing. R Foundation for Statistical Computing, Vienna, Austria. ISBN 3-900051-07-0.
2. Jousselin E, Van Noort S, Berry V, Rasplus JY, Rønsted N, et al. (2008) One fig to bind them all: Host conservatism in a fig wasp community unraveled by cospeciation analyses among pollinating and nonpollinating fig wasps. Evolution 62: 1777-1797.
3. Legendre P, Desdevises Y, Bazin E (2002) A statistical test for host-parasite coevolution. Syst Biol 51: 217-234.
4. Hommola K, Smith JE, Qiu Y, Gilks WR (2009) A permutation test of host-parasite cospeciation. Mol Biol Evol 26: 1457-1468.
5. De Vienne DM, Giraud T, Shykoff JA (2007) When can host shifts produce congruent host and parasite phylogenies? A simulation approach. J Evol Biol 20: 1428-1438.
6. Paradis E (2006) Analysis of Phylogenetics and Evolution with R. New York: Springer. 211 p.
7. Oksanen J (2013) Multivariate analysis of ecological communities in R: vegan tutorial. Available: http://cc.oulu.fi/~jarioksa/opetus/metodi/vegantutor.pdf . Accessed 2013 March 12.
8. Hafner MS, Sudman PD, Villablanca FX, Spradling TA, Demastes JW, et al. (1994) Disparate rates of molecular evolution in cospeciating hosts and parasites. Science 265: 1087-1090.
9. Hafner MS, Demastes JW, Spradling TA, Reed DL (2003) Cophylogeny between pocket gophers and chewing lice. In: Page RDM, editor. Tangled Trees: Phylogeny, Cospeciation and Coevolution. Chicago: University of Chicago Press. pp. 195-220.
10. Felsenstein J, Churchill GA (1996) A Hidden Markov Model approach to variation among sites in rate of evolution. Mol Biol Evol 13: 93-104.
11. Swofford DL (2001) PAUP*. Phylogenetic analysis using parsimony (and other methods). Sinauer Associates.
12. Hasegawa M, Kishino H, Yano T (1985) Dating of the human-ape splitting by a molecular clock of mitochondrial DNA. J Mol Evol 22: 160-174.
13. Legendre P, Legendre L (1998) Numerical Ecology. Amsterdam: Elsevier. 853 p.

Appendix

This appendix illustrates the format of the input files used in the present document. **Files should have matching taxa labels**. The following labels were used:

Pocket gophers: Ccas: *Cratogeomys castanops*; Cmer: *C. merriami*; GburA: *Geomys bursarius halli*; GburB: *G. bursarius majusculus*; Gbre: *G. breviceps*; Gpers: *G. personatus*; Ocav: *Orthogeomys cavator*; Oche: *O. cherriei*; Ohet: *O. heterodus*; Ohis: *O. hispidus*; Ound: *O. underwoodii*; Pbul: *Pappogeomys bulleri*; Ztri: *Zygogeomys trichopus*; Tbot: *Thomomys bottae*; Ttal: *T. talpoides.* **Chewing lice:** Gact: *Geomydoecus actuosi*; Gcha: *G. chapini*; Gche: *G. cherriei*; Gcos: *G. costaricensis*; Gewi; *G. ewingi*; Gexp: *G. expansus*; Ggeo: *G. geomydis*; Gnad: *G. nadleri*; Gokl: *G. oklahomensis*; Gpan: *G. panamensis*; Gpero: *G. perotensis*; Gset: *G. setzeri*; Gtex: *G. texanus*; Gtho: *G. thomomyus*; Gtri: *G. trichopi*; Tbar: *Thomomydoecus barbarae*; Tmin: *T.minor.*

A1. Host-parasite association matrix (g-l_links.txt)

Binary matrix with host and parasite species in rows and columns, respectively; 1's represent presence of a given parasite in a given host in nature, whereas 0's denote otherwise.

	Gset	Gpan	Gche	Gcos	Gcha	Ggeo	Gokl	Gewi	Gtex	Gexp	Gact	Gpero	Gtho	Gtri	Gnad	Tmin	Tbar
GburA	0	0	0	0	0	0	1	0	0	0	0	0	0	0	0	0	0
GburB	0	0	0	0	0	1	0	0	0	0	0	0	0	0	0	0	0
Gbre	0	0	0	0	0	0	0	1	0	0	0	0	0	0	0	0	0
Gpers	0	0	0	0	0	0	0	0	1	0	0	0	0	0	0	0	0
Ound	1	0	0	0	0	0	0	0	0	0	0	0	0	0	0	0	0
Ocav	0	1	0	0	0	0	0	0	0	0	0	0	0	0	0	0	0
Oche	0	0	1	0	0	0	0	0	0	0	0	0	0	0	0	0	0
Ohet	0	0	0	1	0	0	0	0	0	0	0	0	0	0	0	0	0
Ohis	0	0	0	0	1	0	0	0	0	0	0	0	0	0	0	0	0
Pbul	0	0	0	0	0	0	0	0	0	0	0	0	0	0	1	0	0
Ccas	0	0	0	0	0	0	0	0	0	1	0	0	0	0	0	0	0
Cmer	0	0	0	0	0	0	0	0	0	0	0	1	0	0	0	0	0
Ztri	0	0	0	0	0	0	0	0	0	0	0	0	0	1	0	0	0
Tbot	0	0	0	0	0	0	0	0	0	0	1	0	0	0	0	1	0
Ttal	0	0	0	0	0	0	0	0	0	0	0	0	1	0	0	0	1

A2. Tree files

Newick format files with branch lengths and taxon labels.

A2.1 Pylogeny of pocket gophers (phylo_gopher.tre):

```
(Ttal:0.07713,Tbot:0.08101,(((((((Oche:0.02354,Ohet:0.01733):0.01634,(Ocav:0.04239,Ound:0.04272):0.01389):0.01327,Oh
is:0.05788):0.03688,Pbul:0.08235):0.00364,(Cmer:0.04659,Ccas:0.05507):0.03224):0.0047,Ztri:0.10476):0.00323,(Gpers:0
.05442,(Gbre:0.07104,(GburA:0.03003,GburB:0.04034):0.02281):0.01448):0.01172):0.04061);
```

A2.2 Pylogeny of chewing lice (phylo_lice.tre):

```
(Tbar:0.07682,Tmin:0.10403,((((Gtho:0.14298,Gact:0.10454):0.01104,Gpero:0.10711):0.00819,(((Gche:0.07859,Gcos:0.0556
7):0.03793,(Gpan:0.07292,Gset:0.07992):0.01251):0.01527,Gcha:0.10553):0.01919):0.00187,((Gnad:0.11749,Gtri:0.09812):
0.0122,(Gexp:0.11179,((Gtex:0.03366,Gewi:0.03409):0.06301,(Gokl:0.02618,Ggeo:0.02384):0.07929):0.01325):0.01303):0.0
0524):0.0262);
```

A3. Aligned sequences

A3.1 Sequences of pocket gophers (alig_gopher.fasta):

```
>GburA
TGAAGTTTATATCTTAATTCTACCTGGATTCGGAATAATTTCACACATTGTTACTTATTATTCAGGAAAAAAAGAACCTTTTGGCTACATAGGCATAGTTTGAGCTATGATATCAA
TTGGATTCCTAGGCTTTATTGTATGAGCCCATCATATATTTACAGTAGGTATGGATGTAGACACCCGAGCCTATTTTACATCTGCAACTATAATCATTGCTATCCCAACAGGAGTA
AAAGTGTTTAGCTGACTAGCTACTTTACACGGAGGTAATATTAAATGATCTCCTGCTATACTGTGAGCATTAGGCTTTATTTTCCTTTTCACTATTGGCGGATTAACTGGAATCGT
CCTATTCAACTCATCATTAGATATTGTATTA
>GburB
TGAAGTTTACATCTTAATCCTACCCGGATTCGGAATAATTTCCCATATTGTTACCTATTACTCAGGAAAAAAAGAGCCTTTTGGCTACATAGGCATAGTTTGAGCCATGATATCAA
TTGGATTCCTAGGCTTTATTGTTTGAGCCCATCATATATTTACAGTAGGTATGGACGTAGATACCCGAGCCTATTTTACATCTGCAACTATAATCATCGCTATCCCAACAGGAGTA
AAAGTATTCAGCTGATTAGCTACTTTACACGGGGGTAATATTAAATGATCACCTGCTATACTGTGAGCATTAGGCTTTATTTTCCTTTTCACTATTGGCGGGTTAACTGGAATTGT
CCTGTCCAACTCATCATTAGACATTGTTCTA
```

>Gbre
TGAAGTTTATATCTTGATCCTGCCAGGATTTGGAATAATTTCACATATTGTTACTTACTATTCCGGAAAAAAAGAGCCTTTTGGCTATATAGGTATAGTTTGAGCTATAATATCAA
TCGGATTTTTAGGCTTTATCGTGTGAGCCCACCATATATTTACAGTAGGTATGGATGTAGACACACGAGCTTACTTTACATCTGCTACTATAATTATCGCCATCCCAACTGGAGTG
AAAGTATTTAGCTGATTAGCCACTTTACACGGAGGTAATATTAAATGGTCACCTGCTATATTGTGAGCGCTAGGTTTTATTTTCCTTTTCACTATCGGCGGATTAACTGGAATCGT
CCTGTCCAACTCATCACTAGACATTGTACTG
>Gpers
TGAGGTTTACATCCTAATTCTACCAGGATTCGGTATAATTTCACATATTGTCACTTATTATTCAGGAAAAAAGGAACCCTTTGGCTATATAGGCATAGTTTGAGCTATAATATCAA
TTGGGTTCTTAGGTTTTATTGTATGAGCCCACCATATATTTACAGTAGGCATGGACGTAGATACCCGAGCCTACTTTACATCTGCAACTATAATTATCGCTATTCCTACAGGAGTA
AAAGTATTTAGCTGACTAGCCACTCTACACGGAGGTAATATTAAATGATCCCCTGCTATACTATGAGCACTAGGCTTTATTTTCCTTTTCACTATTGGTGGGCTAACTGGTATCGT
CTTATCCAACTCATCATTAGACATCGTACTG
>Ound
TGAAGTTTATATCTTGATCCTCCCAGGATTCGGAATGATTTCTCATATTGTCACCTACTATTCAGGTAAAAAAGAACCCTTTGATTATATAGGCATGGTATGAGCTATAATATCTA
TTGGTTTCCTAGGTTTTATAGTATGAGCCCACCATATATTCACAGTAGGGATAGACGTAGATACACGAGCTTACTTTACATCCGCTACTATGATTATCGCTATCCCTACCGGAGTA
AAAGTGTTCAGTTGACTAGCTACCATGAACGGAGGTAATATTAAATGATCTCCTGCCATATTATGAGCCTTAGGTTTTATTTTCCTGTTCACAATTGGTGGACTAACAGGCATTGT
ATTATCTAATTCATCCTTAGACATTATTCTA
>Ocav
TGAAGTTTATATCTTAATTCTCCCAGGCTTCGGAATGATTTCTCATATTGTCACTTACTACTCAGGTAAAAAAGAACCATTTGATTATATAGGCGTGGTATGAGCTATAATATCCA
TCGGATTCCTAGGTTTGATAGTATGAGCCCACCATATATTCACAGTAGGAATAGACGTAGACACACGAGCTTATTTCACATCCGCTACTATAATTATTGCTATTCCCACCGGAGTA
AAAGTATTCAGTTGACTGGCTACCATGAACGGAGGTAATATTAAATGATCTCCTGCCATACTATGAGCTTTAGGCTTTATCTTCCTATTCACAATTGGTGGCCTAACCGGCATTGT
ATTATCAAATTCATCCTTAGATATTATTCTA
>Oche
TGAAGTTTACATCTTAATCCTCCCAGGCTTCGAAATAATCTCTCATATTGTCACTTATTATTCAGGTAAAAAAGAACCCTTTGGCTATATAGGTATGGTATGAGCTATAATATCAA
TTGGTTTCCTAGGTTTAATGGTATGAGCCCACCATATATTTACAGTAGGAATAGACGTAGATACACGAGCCTACTTTACATCCGCTACTATAATTATTGCTATTCCTACCGGAGTA
AAAGTATTCAGTTGACTAGCTACCATGAACGGAGGCAATATTAAATGATCCCCTGCCATATTATGAGCTTTAGGTTTTATTTTCCTATTTACAATTGGTGGCCTAACCGGCATTGT
ATTATCAAACTCATCCTTAGATATTGTCCTA
>Ohet
TGAAGTTTACATCTTAATCCTCCCAGGCTTCGGAATGATTTCTCATATTGTCACTTATTATTCAGGTAAAAAAGAACCCTTTGGCTATATAGGCATGGTATGAGCTATAATATCAA
TTGGTTTCCTAGGTTTTATGGTATGAGCCCACCATATATTTACAGTAGGGATAGACGTAGATACACGAGCCTACTTTACATCCGCTACTATAATTATTGCTATTCCTACAGGAGTA
AAAGTATTCAGTTGACTGGCTACCATGAACGGAGGTAATATTAAATGATCCCCTGCCATATTATGAGCTTTAGGTTTTATTTTCCTATTTACAATTGGGGGCCTAACCGGCATTGT
ACTATCCAACTCATCATTGGATATTGTTCTA
>Ohis
TGAGGTTTATATCTTAATCCTCCCAGGCTTCGGTATAATTTCTCATATCGTCACTTATTACTCAGGCAAAAAAGAACCATTTGGATATATAGGCATGGTATGAGCCATAATATCTA
TTGGTTTCTTAGGTTTCATGGTATGAGCCCACCATATATTCACAGTAGGAATAGACGTAGACACACGAGCTTATTTTACATCTGCTACTATAATTATCGCTATTCCTACCGGGGTA
AAAGTATTCAGTTGACTGGCTACACTGCATGGAGGTAATATTAAATGATCTCCTGCCATATTATGAGCTTTAGGTTTTATTTTCCTATTTACTATCGGTGGTCTAACGGGCATTGT
CCTATCAAATTCATCTTTGGATATCGTTCTA
>Pbul
TGAAGTTTATATTTTAATTCTCCCAGGTTTCGGAATAATTTCACATATCGTAACCTATTATTCTGGTAAAAAAGAGCCATTCGGTTACATAGGTATAGTATGGGCTATAATATCAA
TTGGCTTCTTAGGATTTATCGTATGAGCCCACCATATATTTACAGTAGGAATGGACGTAGACACACGAGCTTATTTTACATCTGCAACTATAATTATTGCTATCCCCACCGGAGTT
AAAGTCTTTAGCTGACTGGCCACTCTTCATGGTGGTAATATTAAATGATCACCTGCCATACTATGAGCTCTAGGTTTCATTTTCCTATTCACTATTGGAGGCTTAACTGGTATCGT
ACTATCAAATTCATCATTAGATATTGTCTTA
>Ccas

AGAGGTTTATATCTTAATTCTTCCTGGATTTGGTATAATTTCACACATCGTCACTTATTACTCAGGTAAAAAGAACCATTTGGCTATATAGGAATAGTTTGAGCCATAATATCAA
TTGGCTTTTTAGGCTTCATCGTGTGAGCTCACCATATGTTTACGGTAGGGATAGATGTAGACACCCGAGCTTATTTTACATCTGCAACTATAATCATTGCTATTCCTACAGGAGTA
AAGGTATTTAGTTGATTGGCCACCCTCCACGGAGGTAATATTAAATGATCACCAGCCACGTTATGAGCCCTAGGCTTTATTTTCCTTTTTACTATTGGCGGATTAACTGGTATCGT
GTTATCAAACTCATCATTAGATATTGTACTA
>Cmer
AGAAGTCTATATCCTAATCCTCCCTGGATTCGGAATAATTTCACACATCGTCACCTATTATTCAGGTAAGAAAGAACCGTTCGGTTATATAGGAATGGTTTGAGCTATAATATCAA
TTGGCTTTTTAGGTTTCATCGTATGGGCCCACCATATATTCACAGTAGGAATGGACGTAGACACCCGAGCTTATTTTACATCAGCAACTATAATTATTGCTATTCCTACCGGAGTA
AAAGTATTTAGTTGATTGGCCACCCTCCACGGAGGTAATATTAAATGATCACCAGCCACATTATGAGCTCTAGGCTTTATTTTCCTTTTCACTATTGGCGGTTTAACTGGTATCGT
GTTATCAAACTCATCATTAGATATCGTATTA
>Ztri
AGAAGTTTACATTCTAATCCTCCCTGGCTTCGGTATAATCTCACACATTGTCACCTACTATTCAGGCAAAAAAGAACCATTCGGATATATAGGTATAGTATGAGCTATAATGTCCA
TTGGCTTTCTAGGCTTTATCGTATGAGCCCACCATATATTTACGGTAGGTATAGACGTAGATACACGAGCTTACTTCACATCCGCAACAATAATTATCGCCATTCCTACTGGAGTA
AAAGTATTCAGCTGATTAGCTACATTACATGGAGGTAATATCAAATGATCACCCGCTATGCTATGAGCCCTAGGCTTCATTTTTCTTTTCACTATTGGTGGTCTAACAGGTATCGT
CCTATCCAACTCATCACTAGATATCGTCTTA
>Tbot
TGAAGTTTATATCCTAATTCTTCCAGGGTTTGGAATAATTTCCCATATTGTTACCTACTATTCAGGCAAAAAGGAACCTTTCGATTATATGGGAATAGTATGAGCTATAATATCTA
TCGGATTTCTAGGTTTTATTGTTTGAGCACATCATATGTTTACAGTTGGAATAGACGTAGATACTCGAGCCTATTTTACATCTGCTACTATAATTATTGCTATTCCTACCGGTGTA
AAAGTTTTCAGCTGACTTGCTACTCTTCACGGAGGAAACATTAAATGATCACCAGCTATGCTATGAGCATTGGGCTTTATTTTCCTTTTTACAGTTGGAGGTTTAACTGGAATTGT
TTTATCAAACTCATCATTGGACATTGTACTA
>Ttal
AGAAGTTTATATCTTAATTTTACCTGGGTTTGGAATAATTTCCCACATTGTTACTTACTACTCAGGCAAAAAAGAACCTTTTGGGTATATGGGAATAGTATGAGCCATAATATCAA
TCGGGTTTTTAGGGTTTATCGTATGAGCACACCACATGTTTACAGTGGGAATAGATGTAGATACCCGGGCCTACTTTACATCTGCTACAATAATTATTGCTATTCCAACAGGAGTA
AAAGTTTTTAGCTGATTAGCTACCCTTCATGGCGGTAATATTAAATGATCCCCCGCCATACTATGAGCATTAGGTTTTATTTTCCTTTTTACAGTTGGAGGACTGACTGGAATTGT
TCTATCTAACTCATCACTAGATATTGTATTA

A3.2 Sequences of chewing lice (alig_lice.fasta):

>Gset
CGAAGTTTATATTTTGATTTTACCAGGTTTTGGGTTAATTTCTCAAATTATTTTATTCGAGAGAGGAAAGAAGCAAGTTTTTGGAACTGTAGGAATAATCTATGCTATAATAGCAA
TTGGAGTTCTTGGATTCGTGGTTTGAGCTCATCACATATTCACGGTTGGGATGGATGTGGATAGCCGGGCATATTTTACAAGAGCTACGATAGTAATTGCTGTTCCTACTGGAGTT
AAAGTGTTTAGATGATTAGCCACTTCTTTTGGAAGCCGAATTTCATATTCGGTTTCGATACTTTGAACGCTGGGATTCGTATTCCTATTCACCGTGGGAGGGATGACAGGATTGGT
GTTAGCTAATTCGTGCGTAGATGTGGTCCTA
>Gpan
AGAAGTTTATATTTTGATTTTACCTAGATTTGGTTTGATTTCTCAAATTATTCTATTCGAAAGAGGAAAGAAACAGGTCTTTGGAACCGTGGGAATAATTTATGCAATAATAGCGA
TTGGAGTTCTTGGATTTGTGGTTTGGGCTCATCACATATTTACTGTTGGGATAGATGTTGATAGACGAGCATATTTTACGAGAGCTACGATAGTAATTGCCGTTCCAACAGGGGTA
AAGGTGTTTAGGTGATTGGCCACTTCTTTTGGAAGCCGAATTTCATATTCGGTTTCGATGCTCTGAACATTAGGATTCGTATTTCTGTTCACGGTTGGAGGGATAACGGGGTTAGT
TCTAGCCAATTCGTGTGTAGATGTAGTCCTA
>Gche
AGAAGTCTATATTTTGATTTTACCCGGATTTGGTTTAATTTCCCAAATTATTTTATTCGAAAGAGGGAAAAAGCAGGTTTTTGGTACCGTGGGTATAATTTATGCGATAATAGCAA
TCGGAGTATTGGGTTTCGTGGTATGGGCTCATCACATGTTTACCGTTGGAATGGATGTAGATAGCCGAGCATACTTCACAAGAGCAACGATAGTAATTGCTGTTCCAACCGGGGTG

AAAGTATTTAGATGATTAGCCACTTCGTTCGGGAGTCGAGTCTCTTATTCCGTCTCTATACTTTGAACTTTGGGGTTCGTATTCTTGTTTACGGTAGGAGGAATAACAGGGTTAGT
TTTAGCAAACTCATGTGTGGACGTAGTCCTA
>Gcos
CGAAGTTTATATTTTGATTTTACCCGGATTTGGTTTGATTTCTCAGATTATTTTATTTGAAAGAGGAAAGAAGCAGGTTTTTGGAACTGTAGGGATAATTTATGCAATAATAGCAA
TCGGAGTACTAGGTTTCGTGGTATGGGCACACCACATGTTTACCGTTGGGATGGATGTTGACAGTCGAGCGTATTTCACAAGAGCAACGATAGTAATTGCTGTTCCAACTGGGGTA
AAGGTATTTAGATGACTAGCCACTTCGTTTGGAAGCCGGGTCTCTTATTCCGTTTCCATGTTATGAACTCTAGGATTTGTATTCTTATTTACGGTAGGAGGAATAACAGGGTTGGT
TTTAGCAAATTCTTGTGTGGATGTAGTCCTA
>Gcha
CGAGGTATATATTTTAATTTTACCAGGTTTCGGTTTAATTTCCCAGATTATTCTATTCGAAAGGGGAAAGAAACAGGTTTTTGGAACTGTCGGGATGATTTATGCTATAATGGCTA
TTGGTATTCTTGGGTTTGTCGTTTGGGCGCATCACATGTTTACCGTAGGGATGGATGTTGACAGTCGAGCATATTTTACTAGAGCTACAATGGTTATTGCTGTTCCGACAGGAGTA
AAAGTATTCAGTTGATTAGCTACTTCATTTGGAAGTCGGATTTCGTATTCCGTTTCTATTTTGTGAACCCTTGGGTTCGTATTTTTATTTACGGTTGGAGGAATGACAGGATTAGT
TTTGGCTAATTCGTGTGTTGATGTGGTCCTA
>Tmin
CGAGGTTTATATTTTGATCCTTCCTGGATTTGGATTAATCTCCCAGATTATCCTTTATGAAAGAGGAAAGAAACAGGTTTTTGGGACAATTGGAATGATTTACGCTATAATAGCAA
TTGGGGTATTAAGGTTTGTGGTTTGGGCCCATCACATATTTACTGTAGGCATAGATGTAGATAGACGGGCTTATTTCACCAGAGCCACTATGGTAATTGCTGTTCCAACTGGGGTA
AAGGTATTCAGGTGATTGGCCACATCTTTTGGGAGACGGATGTCATATTCTATTTCTATACTCTGGGCAATCGGGTTTGTATTTTTATTCACTGTCGGGGGGATGACTGGTTTAGT
TTTGGCAAATTCTTGTGTGGATGTGGTTTTA
>Tbar
CGAAGTTTATATTTTAATCTTACCAGGGTTTGGTTTAATTTCTCAAATCATTTTGTATGAAAGAGGAAAGAAGCAGGTTTTTGGGACTATTGGGATGATTTATGCTATAATGGCGA
TTGGGGTATTAGGTTTTGTGGTTTGAGCACATCATATATTCACCGTTGGGATAGATGTCGATAGTCGGGCATATTTTACCAGGGCTACTATGGTAATTGCTGTTCCTACTGGAGTA
AAAGTGTTTAGTTGATTAGCTACATCTTTTGGTAGACGAATATCATATTCTATTTCCATGATATGAGCAATTGGGTTTGTATTTTTATTTACCGTTGGAGGGATGACTGGTTTAGT
TTTAGCAAATTCTTGTGTGGATGTGGTTCTT
>Gtri
AGAGGTTTATATTCTTATTCTTCCTGGATTTGGGTTAATTTCCCACATTATTTTATTTGAGAGAGGAAAAAAGCAGGTTTTCGGCACTATTGGGATAATTTATGCTATAATGGCTA
TTGGTGTTCTTGGCTTTGTAGTTTGAGCCCACCATATGTTTACTGTGGGGATAGATGTGGATAGACGAGCTTATTTTACTAGAGCAACAATGGTTATTGCTGTTCCTACTGGAGTA
AAAGTTTTTAGTTGGTTAGCTACTTCTTTTGGTGGACGAATGTCGTACTCAGCTTCTATAATGTGGGCAGTAGGGTTTGTGTTTTTATTCACCGTTGGGGGTATGACTGGGTTGGT
TTTGGCAAATTCTTGTGTAGATGTAGTTCTC
>Gnad
AGAGGTNTATATTCTTATTCTCCCTGGATTTGATTTGATTTCTCACATCATTTTACTTGAAAGAGGAAAGAAACAAGTATTTGGCACTGTGGGGATAATTTATGCTATGATGGCAA
TTGGGGTTCTTGGTTTTGTTGTATGAGCTCATCACATGTTTACGGTTGGGATAGACGTTGATAGACGGGCTTATTTTACAAGGGCAACTATGGTTATTGCAGTACCTACGGGGGTA
AAGGTATTTAGATGGTTAGCTACTTCCTTTGGGAGGCGAATAACTTATTCGTCTTCGATGATATGAGCGATTGGGTTCGTATTTTTATTCACTGTTGGGGGCATGACCGGGTTAGT
TTTAGCAAATTCGTGTGTAGACGTTGTATTA
>Ggeo
AGAAGTATACATTCTAATCCTTCCGGAATTTGGTTTAATTTCTCATATTGTATTATTGGAAAGGGGAAAAAAGCAGGTTTTTGGTACTGTTGGGATAATTTACGCAATAATAGCAA
TTGGAGTTCTTGGTTTTGTAGTCTGAGCTCACCACATATTTACTGTAGGAATGGATGTTGATAGCCGGGCATATTTTACTAGGGCGACTATGGTAATTGCGGTACCTACTGGGGTA
AAGGTTTTTAGATGATTGGCCACTTCTTTCGGGAGGCGAATTTCATACTCCGTTTCGACACTGTGGGCATTTGGTTTCATTTTCCTATTTACTATTGGTGGGATAACAGGTTTAGT
TTTAGCAAATTCATGTGTGGATGTGGTCCTA
>Gokl
AGAAGTATACATTCTAATTCTTCCGGGATTCGGGTTAATCTCTCATATTGTATTATTGGAAAGAGGAAAAAAGCAGGTTTTTGGTACTGTTGGGATAATTTACGCAATAATAGCAA
TTGGAGTTCTTGGTTTTGTAGTCTGAGCTCACCATATATTTACTGTAGGAATAGATGTTGATAGCCGGGCATACTTTACTAGGGCGACTATGGTAATTGCGGTGCCTACTGGAGTA
AAGGTTTTTAGGTGATTGGCCACCTCTTTCGGGAGGCGAATTTCATATTCCGTTTCGACACTGTGGGCATTTGGTTTTATTTTTCTATTTACTATTGGTGGGATAACAGGTTTAGT
TCTAGCAAATTCATGTGTAGATGTGGTCCTA

```
>Gewi
TGAAGTTTATATTCTGATTCTTCCAGGATTTGGGTTAATCTCCCACATTGTCCTGTTAGAAAGAGGAAAGAAACAAGTCTTTGGCACTGTCGGAATAATTTATGCAATAATAGCAA
TTGGGGTCCTTGGTTTTGTAGTTTGAGCCCATCACATGTTTACTGTGGGAATGGATGTTGATAGCCGAGCATATTTTACTAGAGCGACTATAGTAATTGCGGTGCCTACCGGAGTA
AAGGTCTTTAGGTGGCTTGCTACTTCTTTTGGAAGTCGGATTTCGTACTCCGTATCGATGCTATGGTCGTTTGGCTTTATTTTTTTATTTACTATCGGCGGCATGACAGGATTAGT
TTTAGCAAATTCTTGTGTAGATGTGGTATTA
>Gtex
TGAAGTTTATATTTTGATCCTTCCAGGATTTGGATTAATCTCCCACATTGTCCTGTTAGAAAGAGGAAAGAAACAAGTCTTTGGTACTGTCGGAATAATTTATGCAATAATAGCAA
TTGGGGTTCTTGGTTTTGTAGTTTGAGCCCACCACATGTTTACTGTAGGGATAGATGTTGATAGCCGGGCATATTTTACTAGAGCTACCATGGTAATTGCGGTACCTACTGGAGTA
AAGGTCTTTAGGTGGCTTGCTACTTCTTTTGGAAGTCGGATTTCGTATTCCGTATCAATACTATGGTCATTTGGTTTTATTTTTTTATTTACTATCGGCGGCATGACAGGATTAGT
TTTAGCAAATTCTCGAGTAGACGTGGTACTA
>Gexp
AGAAGTTTACATTTTGATTCTTCCAGAATTCGGTTTAATTTCCCATATTATTTTACTTGAGAGAGGAAAGAAACAAGTTTTTGGGACTGTTGGGATGATTTATGCGATAATGGCGA
TTGGAGTTTTAGGGTTTGTAGTCTGAGCTCACCACATATTTACTGTTGGGATAGATGTTGACAGTCGGGCATATTTTACCAGGGCAACCATGGTGATTGCAGTGCCCACTGGGGTT
AAAGTTTTTAGGTGGTTGGCTACTTCTTTTGGTAGTCGACTTTCGTATTCAATTTCGACACTTTGGGCATTTGGGTTTATTTTCTTATTTACTATTAGGGGTACGACTGGATTAGT
TTTGGCGAATTCGTGTGTAGATGTAGTCCTA
>Gpero
AGAAGTATATATTTTAATTTTACCCGGATTTGGATTAATTTCCCAAATTATTTTATTTGAGAGAGGAAAAAAACAAGTTTTCGGGACAATTGGTATAATTTATGCAATAATGGCAA
TTGGTGTTTTAGGTTTTGTGGTATGGGCTCATCATATATTTACTGTAGGTATGGATGTAGACAGTCGTGCATATTTTACTAGGGCTACAATAGTAATTGCGGTTCCAACTGGTGTA
AAGGTTTTTAGGTGGCTAGCTACATCTTTTGGAAGTCGAATATCGTTTACAACATCAATGTTGTGGGCGATTGGATTTGTATTTTTATTTACTGTAGGAGGGATAACAGGTTTAGT
TTTAGCTAATTCCTGTGTAGATGTGGTTTTA
>Gtho
TGAAGTGTATATTTTAATTCTACCAGGATTTGGTTTGATTTCTCGAATCATTTTGTTCGAAAGAGCAAAAAAACAAGTGTTCGGTACAGTAGGCATAATTTATGCTATAACTGCTA
TTGGAGTATTGGGGTTTGTGGTGTGAGCCCATCACATGTTTACTGTTGGTATAGATGTAGATAGTCGAGCATACTTTACTAGAGCAACTATAGTTATTGCTGTCCCTACTGGGGTA
AAAGTATTTAGATGGCTTGCTACATCTTTTGGCAGCCGAGTGTCTTACTCTGTCTCTCTTTTATGATCTTCTGGATTTGTATTCCTATTTACTGTAGGAGGAATAACTGGTTTGGT
ATTAGCTAATTCATGTGTAGATGTAATTTTA
>Gact
CGAAATGTATATTTTAATCCTACCAGGATTCGGTTTAATTTCTCACATTATTTTATTAGAAAGAGGAAAAAAACAGGTTTTTGGCACTGTTGGTATAATTTATGCGATAATGGCTA
TTGGTGTATTGGGATTTGTGGTTTGAGCTCACCACATATTCACTGTTGGAATAGATGTAGATAGTCGAGCGTATTTTACTAGGGCAACTATAGTAATTGCGGTACCTACTGGGGTA
AAAGTTTTTAGGTGACTTGCTACCTCCTTTGGAAGTCGAATTTCCTTCTCCGTTTCGATAATATGGGCCTTCGGCTTTATTTTTTTGTTTACTATTGGCGGTATAACGGGCTTAGT
GTTGGCAAATTCATGTGTAGACGTCGTCTTC
```

A4. Genetic distances

A4.1 HKY85 distance matrix of pocket gophers (dist_gopher.txt):

	GburA	GburB	Gbre	Gpers	Ound	Ocav	Oche	Ohet	Ohis	Pbul	Ccas	Cmer	Ztri	Tbot	Ttal
GburA	0	0.07036	0.12782	0.115	0.20408	0.20273	0.19576	0.17476	0.21425	0.15956	0.16259	0.17248	0.20074	0.19232	0.17722
GburB	0.07036	0	0.13025	0.12348	0.19983	0.21755	0.19538	0.1709	0.20616	0.18745	0.19825	0.20861	0.20414	0.18526	0.20606
Gbre	0.12782	0.13025	0	0.14761	0.20842	0.24667	0.21918	0.20787	0.2265	0.1779	0.18249	0.1972	0.19659	0.22756	0.20515
Gpers	0.115	0.12348	0.14761	0	0.17846	0.19938	0.15333	0.14377	0.17094	0.17019	0.15947	0.15104	0.16814	0.18461	0.20125
Ound	0.20408	0.19983	0.20842	0.17846	0	0.08511	0.0948	0.08823	0.13279	0.20057	0.23937	0.19607	0.21642	0.20669	0.23728
Ocav	0.20273	0.21755	0.24667	0.19938	0.08511	0	0.09557	0.09427	0.11633	0.1757	0.21863	0.18528	0.22771	0.19941	0.23171
Oche	0.19576	0.19538	0.21918	0.15333	0.0948	0.09557	0	0.04087	0.11613	0.17989	0.20765	0.17847	0.19638	0.19917	0.20537
Ohet	0.17476	0.1709	0.20787	0.14377	0.08823	0.09427	0.04087	0	0.10577	0.18019	0.19657	0.18165	0.20042	0.19946	0.19587
Ohis	0.21425	0.20616	0.2265	0.17094	0.13279	0.11633	0.11613	0.10577	0	0.17213	0.18182	0.16737	0.2097	0.21779	0.22399
Pbul	0.15956	0.18745	0.1779	0.17019	0.20057	0.1757	0.17989	0.18019	0.17213	0	0.1868	0.14119	0.19351	0.21842	0.22155
Ccas	0.16259	0.19825	0.18249	0.15947	0.23937	0.21863	0.20765	0.19657	0.18182	0.1868	0	0.10166	0.20622	0.21578	0.18191
Cmer	0.17248	0.20861	0.1972	0.15104	0.19607	0.18528	0.17847	0.18165	0.16737	0.14119	0.10166	0	0.17967	0.2126	0.22256
Ztri	0.20074	0.20414	0.19659	0.16814	0.21642	0.22771	0.19638	0.20042	0.2097	0.19351	0.20622	0.17967	0	0.23958	0.22094
Tbot	0.19232	0.18526	0.22756	0.18461	0.20669	0.19941	0.19917	0.19946	0.21779	0.21842	0.21578	0.2126	0.23958	0	0.15814
Ttal	0.17722	0.20606	0.20515	0.20125	0.23728	0.23171	0.20537	0.19587	0.22399	0.22155	0.18191	0.22256	0.22094	0.15814	0

A4.2 HKY85 distance matrix of chewing lice (dist_lice.txt):

	Gset	Gpan	Gche	Gcos	Gcha	Tmin	Tbar	Gtri	Gnad	Ggeo	Gokl	Gewi	Gtex	Gexp	Gpero	Gtho	Gact
Gset	0	0.15285	0.20774	0.18508	0.20672	0.28637	0.20558	0.25492	0.25773	0.23895	0.25154	0.24683	0.24684	0.26487	0.25636	0.2713	0.27781
Gpan	0.15285	0	0.20307	0.18012	0.21644	0.24089	0.23687	0.26877	0.23214	0.23289	0.22842	0.23888	0.24289	0.26214	0.24513	0.26842	0.25225
Gche	0.20774	0.20307	0	0.13426	0.24437	0.28942	0.27768	0.31042	0.28377	0.24705	0.27571	0.2955	0.30766	0.30526	0.26057	0.24025	0.26306
Gcos	0.18508	0.18012	0.13426	0	0.20368	0.26451	0.22371	0.27052	0.25355	0.25926	0.28869	0.25906	0.26638	0.2914	0.23581	0.23521	0.27029
Gcha	0.20672	0.21644	0.24437	0.20368	0	0.25833	0.21789	0.22702	0.26045	0.27646	0.28893	0.24521	0.24124	0.25057	0.23825	0.29333	0.26947
Tmin	0.28637	0.24089	0.28942	0.26451	0.25833	0	0.18085	0.23405	0.27406	0.26303	0.25934	0.24557	0.22602	0.26036	0.24257	0.31228	0.2839
Tbar	0.20558	0.23687	0.27768	0.22371	0.21789	0.18085	0	0.21219	0.23126	0.25821	0.25332	0.28135	0.25983	0.2348	0.20537	0.26172	0.23509
Gtri	0.25492	0.26877	0.31042	0.27052	0.22702	0.23405	0.21219	0	0.21561	0.26088	0.2447	0.24405	0.24713	0.22154	0.2393	0.28044	0.24128
Gnad	0.25773	0.23214	0.28377	0.25355	0.26045	0.27406	0.23126	0.21561	0	0.26134	0.27782	0.2428	0.25414	0.25364	0.26446	0.28466	0.26895
Ggeo	0.23895	0.23289	0.24705	0.25926	0.27646	0.26303	0.25821	0.26088	0.26134	0	0.05002	0.21445	0.20119	0.20727	0.26013	0.31126	0.22115
Gokl	0.25154	0.22842	0.27571	0.28869	0.28893	0.25934	0.25332	0.2447	0.27782	0.05002	0	0.19903	0.19007	0.20666	0.24403	0.3197	0.22055
Gewi	0.24683	0.23888	0.2955	0.25906	0.24521	0.24557	0.28135	0.24405	0.2428	0.21445	0.19903	0	0.06775	0.25981	0.2385	0.26712	0.21988
Gtex	0.24684	0.24289	0.30766	0.26638	0.24124	0.22602	0.25983	0.24713	0.25414	0.20119	0.19007	0.06775	0	0.22878	0.24655	0.29137	0.23083
Gexp	0.26487	0.26214	0.30526	0.2914	0.25057	0.26036	0.2348	0.22154	0.25364	0.20727	0.20666	0.25981	0.22878	0	0.24703	0.31316	0.22625
Gpero	0.25636	0.24513	0.26057	0.23581	0.23825	0.24257	0.20537	0.2393	0.26446	0.26013	0.24403	0.2385	0.24655	0.24703	0	0.24623	0.2376
Gtho	0.2713	0.26842	0.24025	0.23521	0.29333	0.31228	0.26172	0.28044	0.28466	0.31126	0.3197	0.26712	0.29137	0.31316	0.24623	0	0.24752
Gact	0.27781	0.25225	0.26306	0.27029	0.26947	0.2839	0.23509	0.24128	0.26895	0.22115	0.22055	0.21988	0.23083	0.22625	0.2376	0.24752	0
