# Supplementary material for: PACo: A Novel Procrustes Application to Cophylogenetic Analysis
Source: PLoS One. 2013 Apr 8;8(4):e61048. doi: 10.1371/journal.pone.0061048 (PMC3620278; doi:10.1371/journal.pone.0061048)
Supplement: File S2 — Methodological details and additional results. This file includes details about the phylogenetic methods used and plots of empirical cumulative distribution function of the P values obtained in simulations, showing the correctness of the Type I errors of the tests compared for any for any significance level. (PDF) [file pone.0061048.s002.pdf]

File S2 – Supporting information for

PACo: a novel Procrustes application to cophylogenetic analysis

Methodological details and additional results

Juan Antonio Balbuena[1]*, Raúl Míguez-Lozano[1], Isabel Blasco-Costa[2]

[1] Cavanilles Institute of Biodiversity and Evolutionary Biology, University of Valencia, Valencia, Spain.

[2] Institute of Parasitology, Academy Sciences of the Czech Republic, České Budějovice, Czech Republic.

*E-mail: j.a.balbuena@uv.es

Phylogenetic methods

Pocket gophers and chewing lice

The phylogenies of pocket gophers and their chewing lice were based on the mitochondrial cytochrome oxidase I sequences of Hafner et al. [1]. We used the HKY85 substitution model [2] to compute the genetic distances between taxa and the phylogenetic trees for gopher and lice were built by the neighbour-joining method [3] using PAUP* [4]. Although there are probably more appropriate substitution models and phylogenetic methods for the data at hand, our goal was not to reconstruct the most accurate phylogenies possible but to obtain in a rapid and convenient fashion reliable genealogies for the present demonstration. In fact, the phylograms obtained were congruent with the phylogenetic information available and the topological differences concerned species that also showed variable positions in previous work [1,5-8].

Freshwater fishes and **Dactylogyrus** *spp.*

We used Tree Snatcher Plus [9] to estimate the patristic distances from Figure 2 of Šimková et al. [10] depicting the phylogeny of *Dactylogyrus* spp. The patristic distances between fish species were obtained from a new phylogeny built by Maximum Likelihood (ML). GenBank sequences for the Cytochrome b of the fishes used in Šimková et al. [10], together with a new sequence representing *Romanogobio albipinnatus* (GenBank Accession No. EF427401) were aligned using MUSCLE implemented in MEGA v. 5 [11] with default settings. The alignment included 28 taxa and 1,140 nucleotide positions, although two sequences (*R. albipinnatus* and *Perca flavescens*) were shorter. The ML analyses were performed in PhyML 3.0 [12] under the evolutionary model GTR+Γ (general time-reversible model including gamma distributed among-site rate variation), using a nearest-neighbour interchange, tree re-arrangement search strategy and a non-parametric bootstrap validation based on 1,000 replicates. The resulting ML phylogenetic hypothesis (Fig. A) agreed well with that depicted in Šimková et al. [10] except

for a few taxa with unsupported placement in both Šimková et al. [10] and the reconstructed tree here.

+

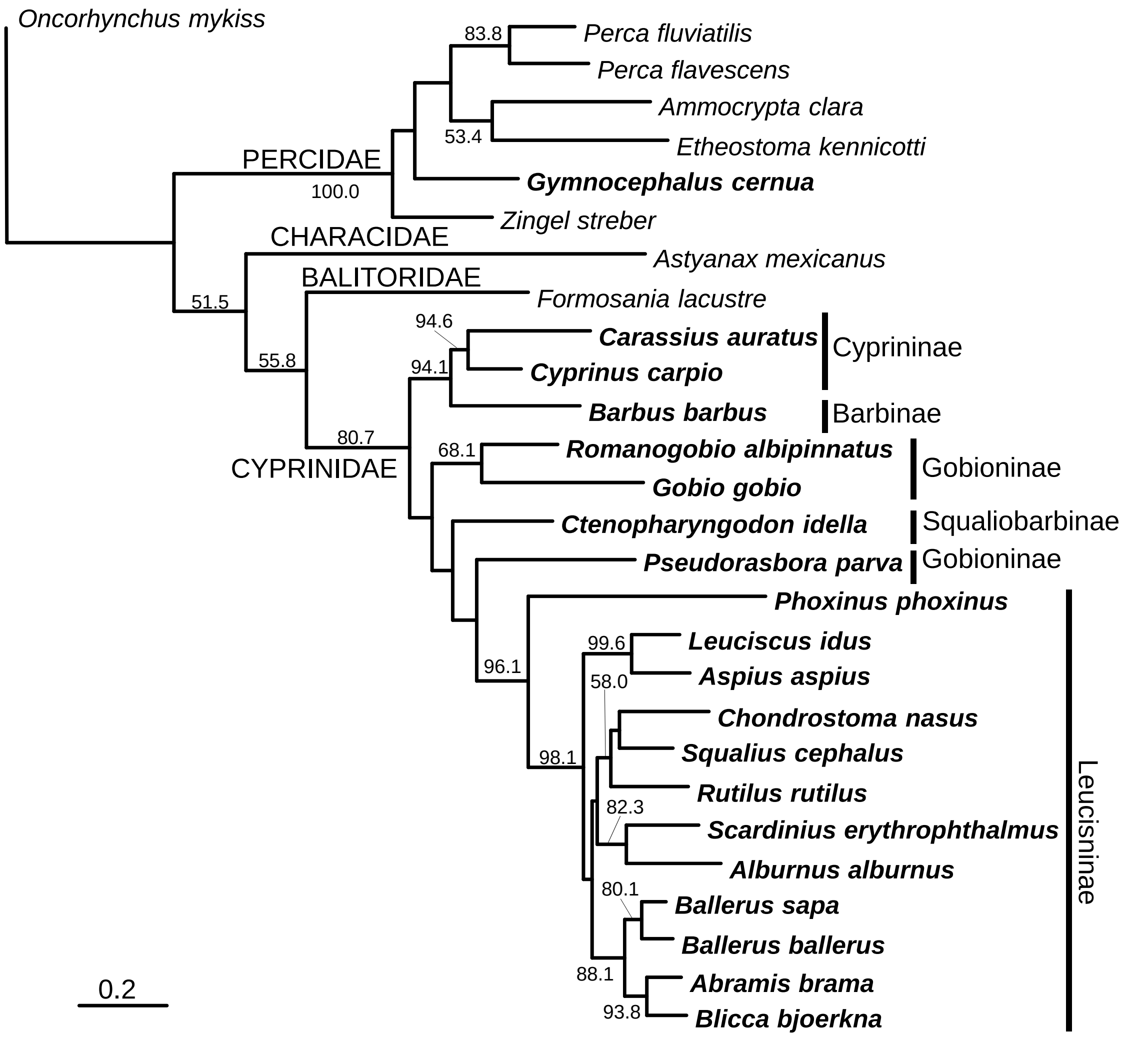

Figure A. Maximum-likelihood tree inferred from Cytochrome b sequences of 28 fish species. The sequence of *Oncorhynchus mykiss* (Salmonidae) was used as outgroup. Species associated to the *Dactylogyrus* spp. studied are boldfaced. Numbers along branches indicate percentages of bootstrap support. (Values < 50% are omitted). Allocation of cyprinid species to subfamilies follow Nelson [13].

Plots of empirical cumulative distribution functions

To evaluate the overall accuracy of the Type I error rates of PACo, Parafit [5] and Hommola et al. Cospeciation Test (HCT) [6], the empirical cumulative distribution function of the *P*-values obtained in simulations with 16 different parameter combinations are plotted in the following pages. The title of each plot identifies the type of test (PACo, ParaFit or HCT) and the parameter combination used, as numbers preceding H, P and L indicate the number of hosts, parasites and links employed in the simulation. The expected cumulative distribution is displayed in red.

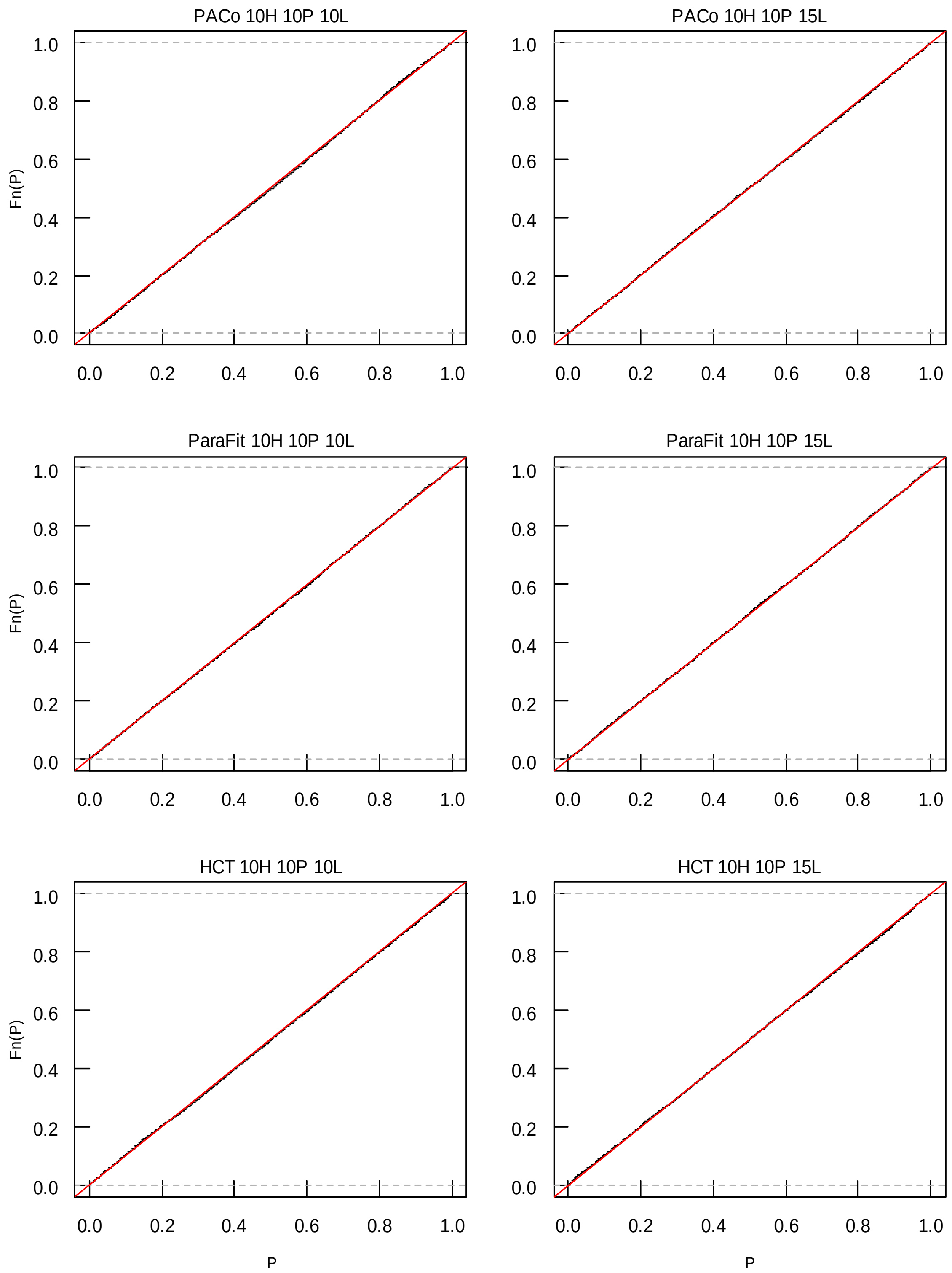
PACo 10H 10P 10L
PACo 10H 10P 15L
ParaFit 10H 10P 10L
ParaFit 10H 10P 15L
HCT 10H 10P 10L
HCT 10H 10P 15L
Fn(P)
P
0.0
0.2
0.4
0.6
0.8
1.0

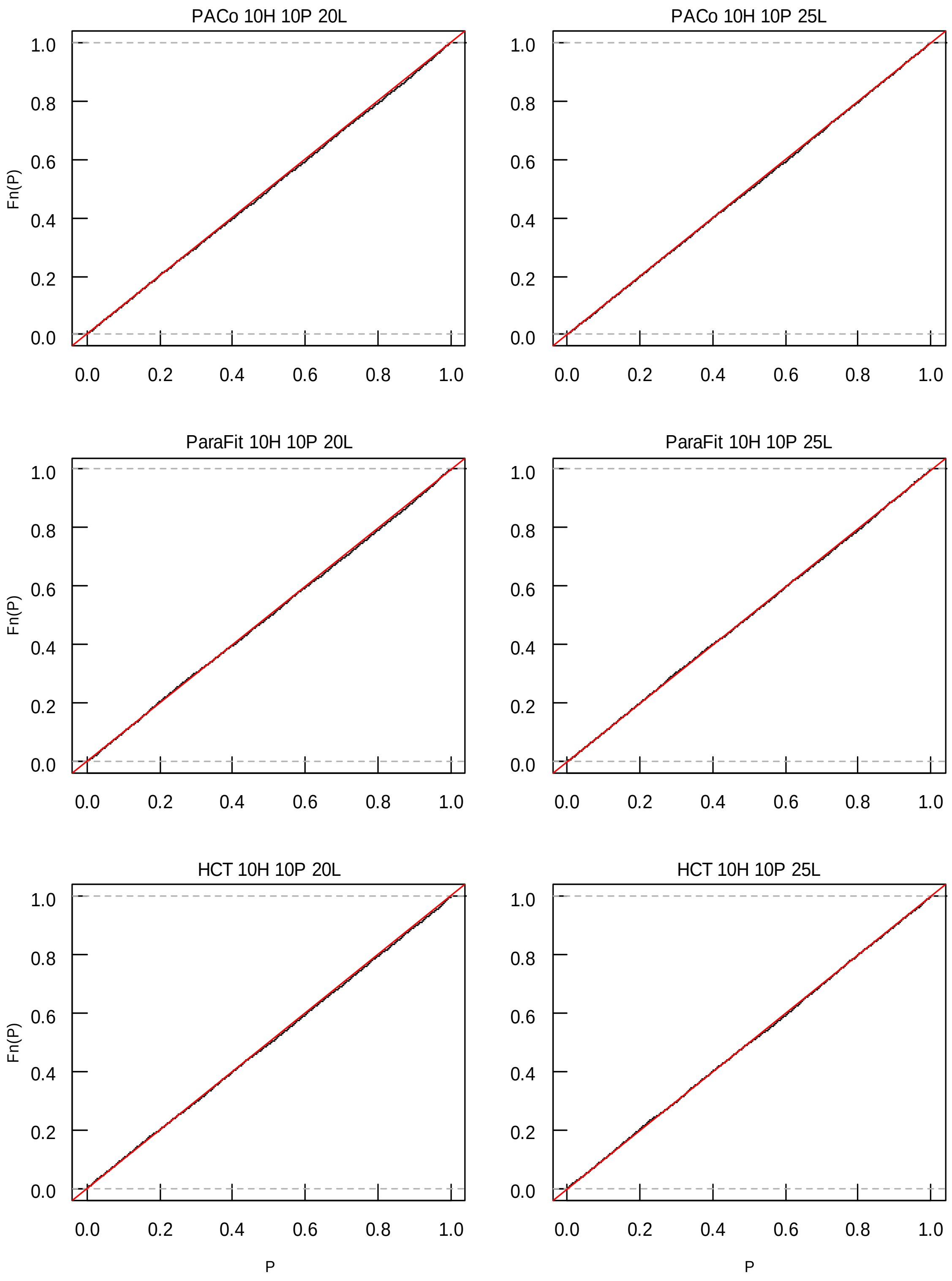
PACo 10H 10P 20L
PACo 10H 10P 25L
ParaFit 10H 10P 20L
ParaFit 10H 10P 25L
HCT 10H 10P 20L
HCT 10H 10P 25L
Fn(P)
P
0.0
0.2
0.4
0.6
0.8
1.0

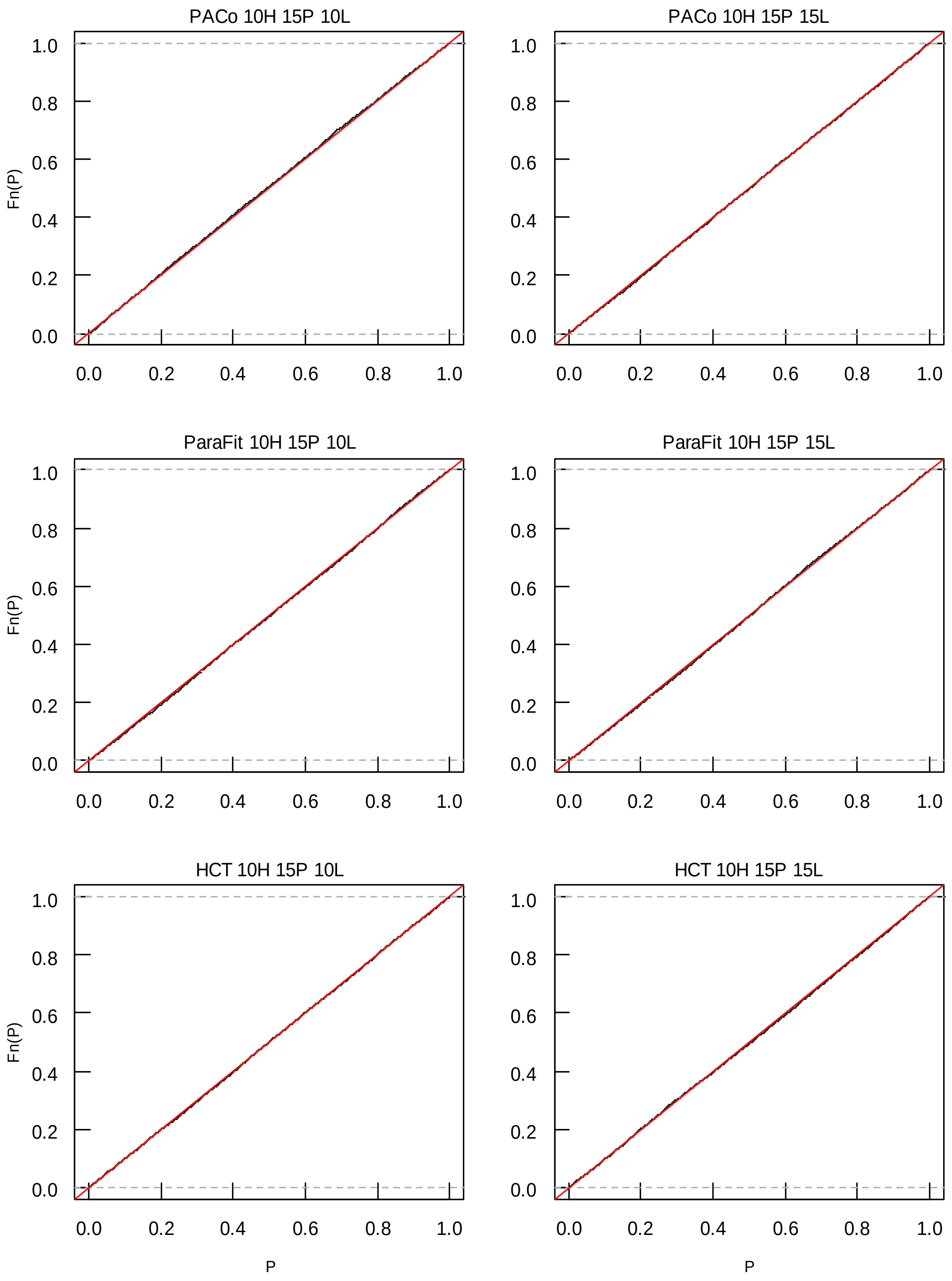
PACo 10H 15P 10L
PACo 10H 15P 15L
ParaFit 10H 15P 10L
ParaFit 10H 15P 15L
HCT 10H 15P 10L
HCT 10H 15P 15L
Fn(P)
P
0.0
0.2
0.4
0.6
0.8
1.0

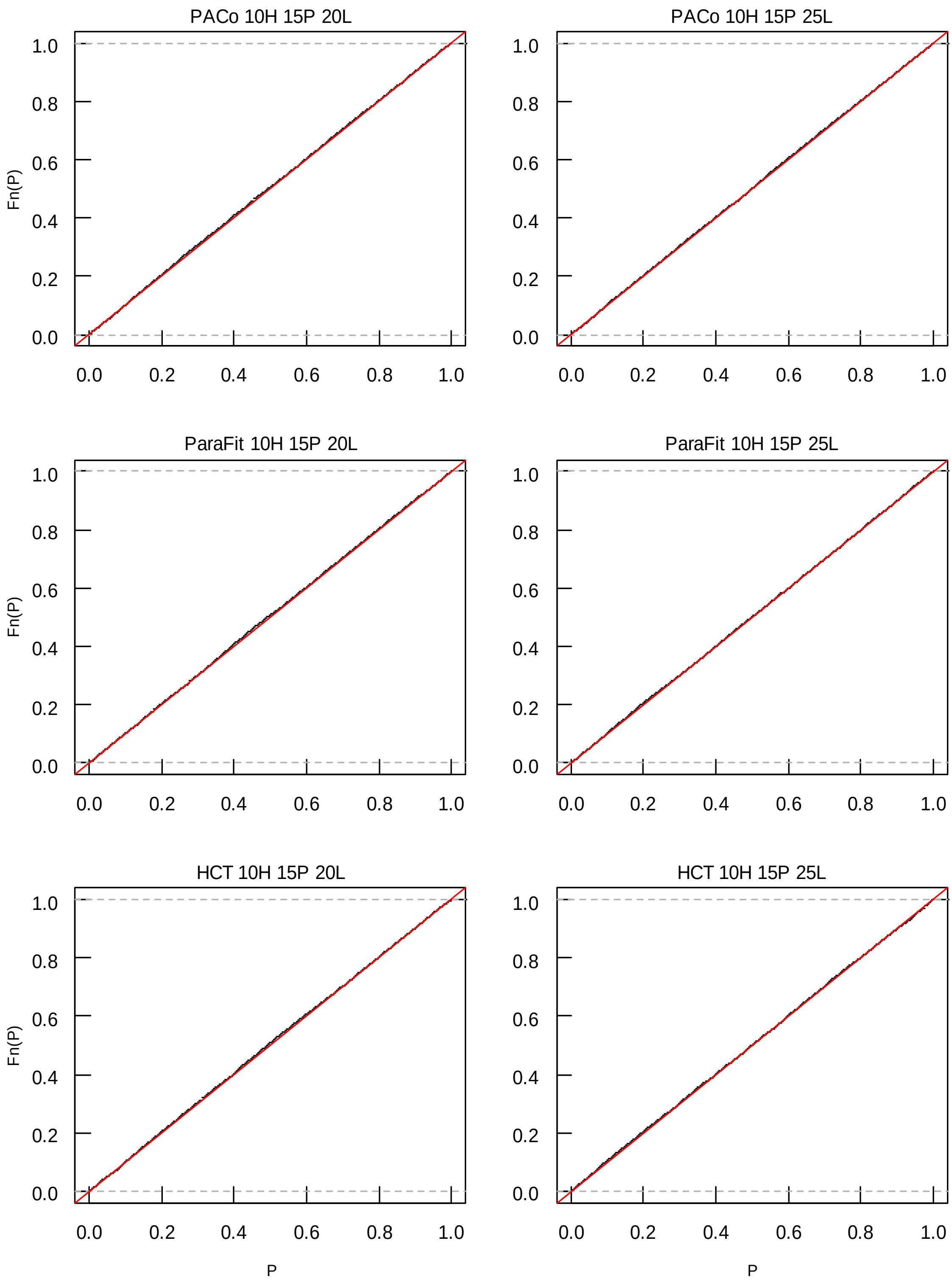
PACo 10H 15P 20L
PACo 10H 15P 25L
ParaFit 10H 15P 20L
ParaFit 10H 15P 25L
HCT 10H 15P 20L
HCT 10H 15P 25L
Fn(P)
P
0.0
0.2
0.4
0.6
0.8
1.0

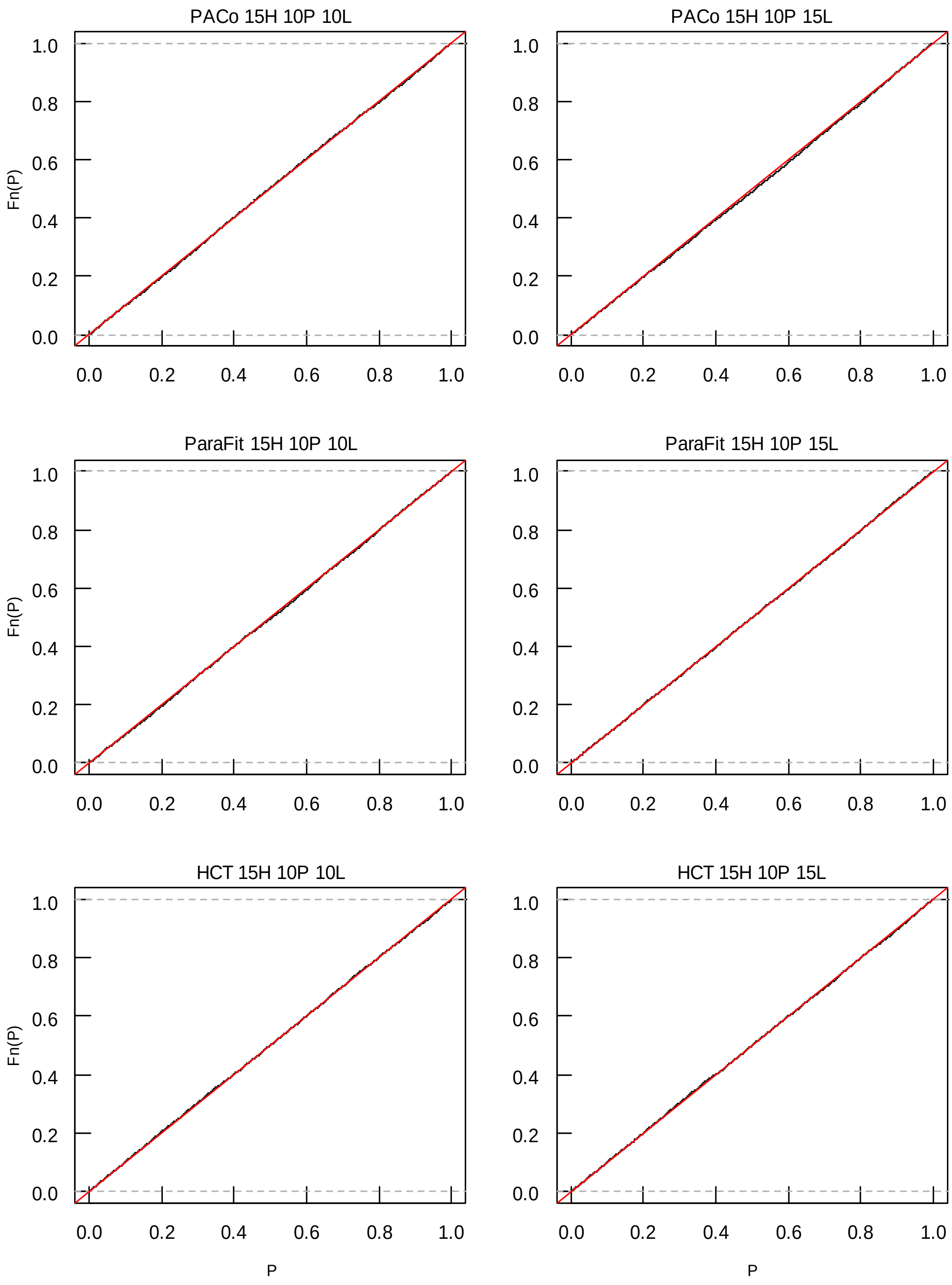
PACo 15H 10P 10L
PACo 15H 10P 15L
ParaFit 15H 10P 10L
ParaFit 15H 10P 15L
HCT 15H 10P 10L
HCT 15H 10P 15L
Fn(P)
1.0
0.8
0.6
0.4
0.2
0.0
P

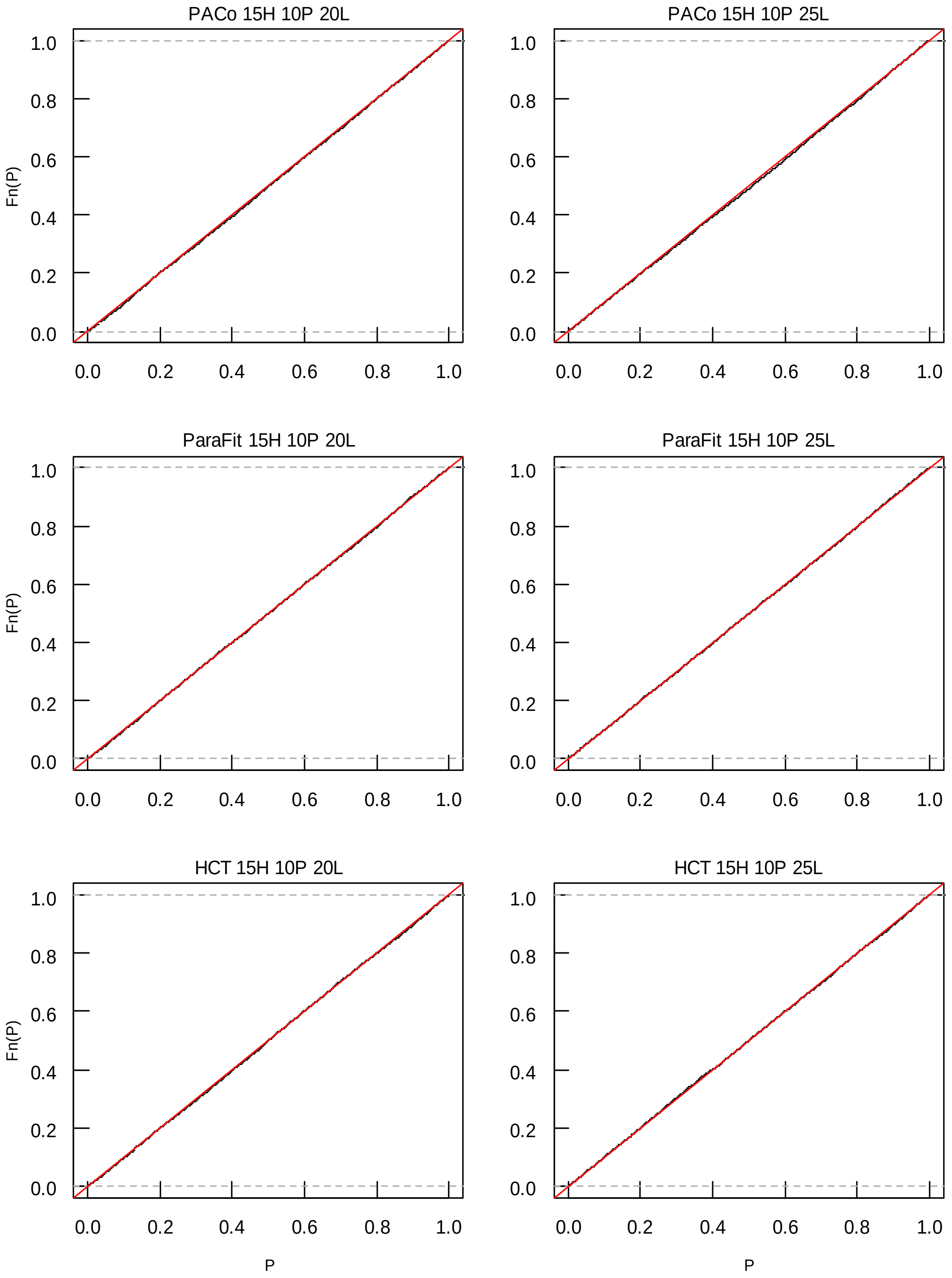
PACo 15H 10P 20L
PACo 15H 10P 25L
ParaFit 15H 10P 20L
ParaFit 15H 10P 25L
HCT 15H 10P 20L
HCT 15H 10P 25L
Fn(P)
P
0.0
0.2
0.4
0.6
0.8
1.0

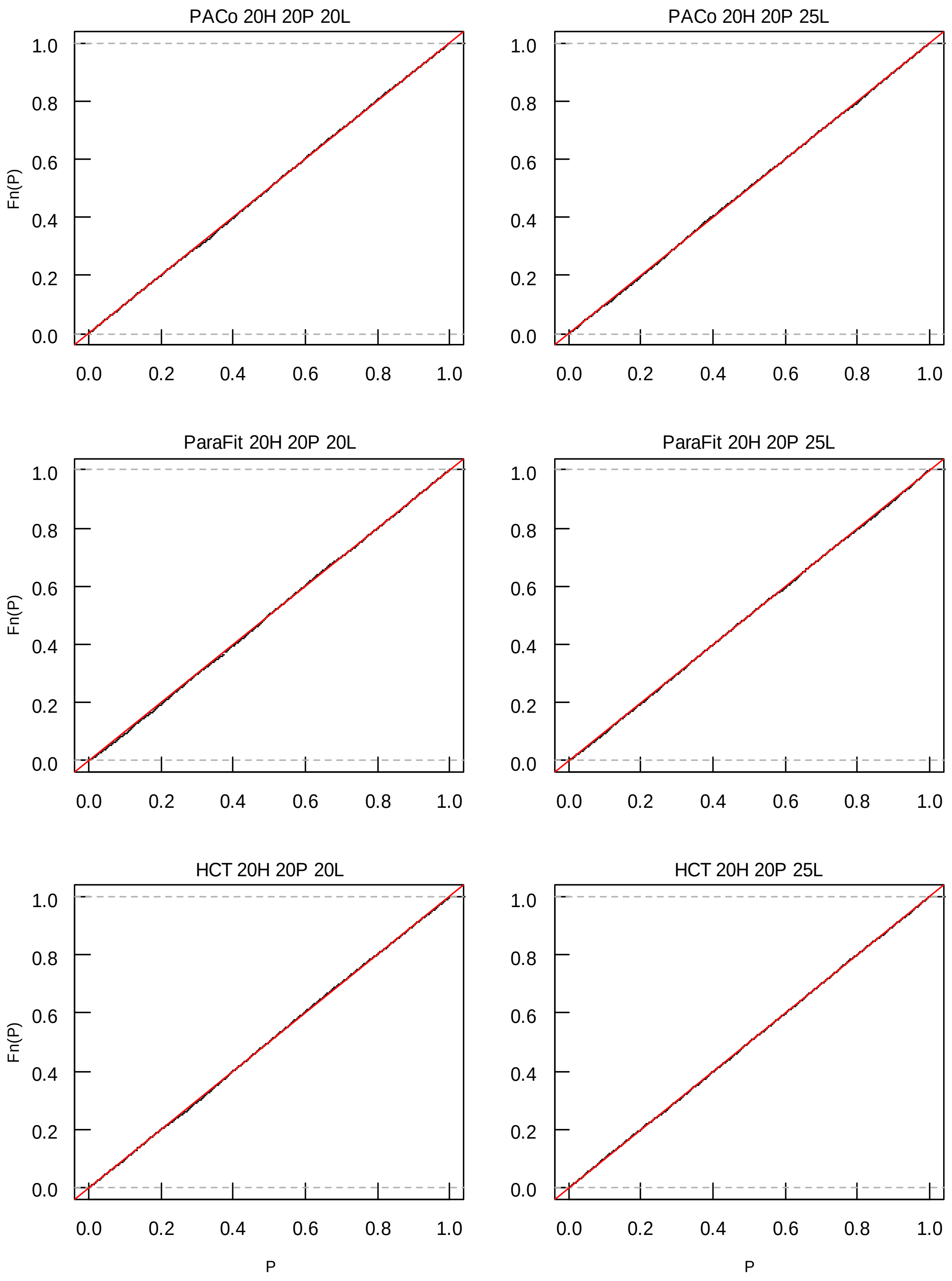
PACo 20H 20P 20L
PACo 20H 20P 25L
ParaFit 20H 20P 20L
ParaFit 20H 20P 25L
HCT 20H 20P 20L
HCT 20H 20P 25L
Fn(P)
P
0.0
0.2
0.4
0.6
0.8
1.0

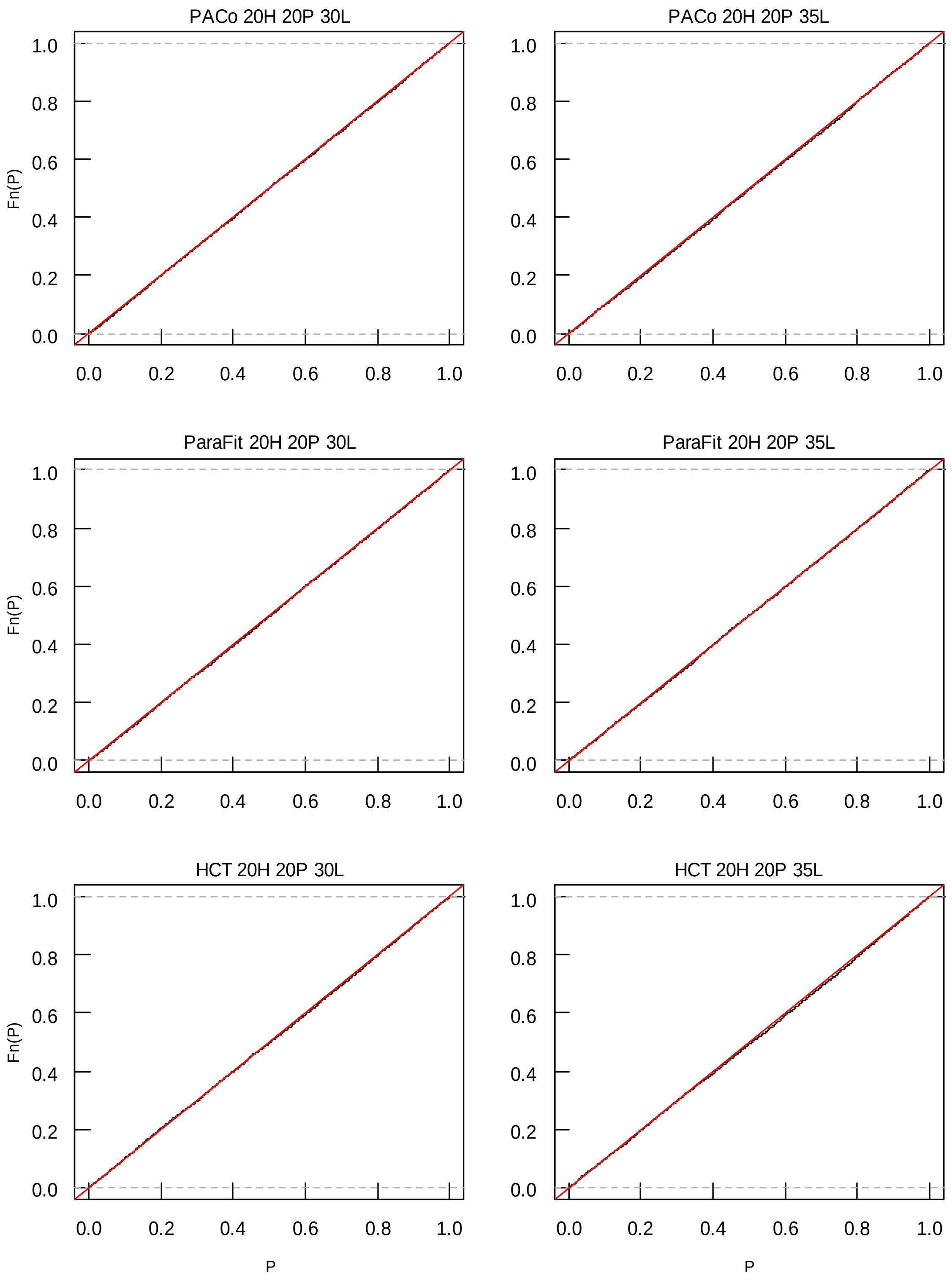

References

1. Hafner MS, Sudman PD, Villablanca FX, Spradling TA, Demastes JW, et al. (1994) Disparate rates of molecular evolution in cospeciating hosts and parasites. Science 265: 1087-1090.

2. Hasegawa M, Kishino H, Yano T (1985) Dating of the human-ape splitting by a molecular clock of mitochondrial DNA. J. Mol. Evol. 22: 160-174.

3. Saitou N, Nei M (1987) The neighbor-joining method: a new method for reconstructing phylogenetic trees. Mol. Biol. Evol. 4: 406-425.

4. Swofford DL (2001) PAUP*. Phylogenetic analysis using parsimony (and other methods). Sinauer Associates.
5. Legendre P, Desdevises Y, Bazin E (2002) A statistical test for host-parasite coevolution. Syst. Biol. 51: 217-234.
6. Hommola K, Smith JE, Qiu Y, Gilks WR (2009) A permutation test of host-parasite cospeciation. Mol. Biol. Evol. 26: 1457-1468.
7. Huelsenbeck JP, Rannala B, Larget B (2003) A statistical perspective for reconstructing the history of host-parasite associations. In: Page RDM, editor. Tangled Trees: Phylogeny, Cospeciation, and Coevolution. Chicago: The University of Chicago Press. pp. 93-119.
8. Hafner MS, Page RDM (1995) Molecular phylogenies and host-parasite cospeciation: gophers and lice as a model system. Phil. Trans. R. Soc. B 349: 77-83.
9. Laubach T, Von haeseler A (2007) TreeSnatcher: Coding trees from images. Bioinformatics 23: 3384-3385.
10. Šimková A, Morand S, Jobet E, Gelnar M, Verneau O (2004) Molecular phylogeny of congeneric monogenean parasites (*Dactylogyrus*): A case of intrahost speciation. Evolution 58: 1001-1018.
11. Tamura K, Peterson D, Peterson N, Stecher G, Nei M, et al. (2011) MEGA5: Molecular evolutionary genetics analysis using maximum likelihood, evolutionary distance, and maximum parsimony methods. Mol. Biol. Evol. 28: 2731-2739.
12. Guindon S, Dufayard JF, Lefort V, Anisimova M, Hordijk W, et al. (2010) New algorithms and methods to estimate maximum-likelihood phylogenies: Assessing the performance of PhyML 3.0. Syst. Biol. 59: 307-321.
13. Nelson JS (2006) Fishes of the world. New York: John Wiley and Sons, Inc. 601 p.
